# Unconventional Biexciton Dynamics in Superradiant Perovskite Quantum Dots Driven by Many‐Body Correlations

**DOI:** 10.1002/adma.74849

**Published:** 2026-08-31

**Authors:** Chenglian Zhu, Jose L. Movilla, Petra Hoffmann, Ihor Cherniukh, Willem P. A. Verheijen, Leon G. Feld, Josep Planelles, Maryna I. Bodnarchuk, Simon C. Boehme, Juan I. Climente, Maksym V. Kovalenko, Gabriele Rainò

**Affiliations:** ^1^ Institute of Inorganic Chemistry Department of Chemistry and Applied Biosciences ETH Zürich Zürich Switzerland; ^2^ Laboratory for Thin Films and Photovoltaics Empa – Swiss Federal Laboratories for Materials Science and Technology Dübendorf Switzerland; ^3^ Departament d'Educacio i Didactiques Especifiques Universitat Jaume I Castello Spain; ^4^ Departament de Química Física i Analítica Universitat Jaume I Castelló Spain

**Keywords:** biexciton, condensed matter physics, coulomb, exciton, photoluminescence, physics, quantum dot, quantum yield, superradiance

## Abstract

Excitons and biexcitons emission traits, for example, quantum yield, lifetime, and coherence, govern device performance metrics including brightness, optical gain thresholds, and the efficiency of single‐ and entangled‐photon generation. Biexcitons are conventionally understood to radiatively decay more rapidly than excitons. This view stems largely from studies in strongly confined quantum dots (QDs), where inter‐exciton correlations are often neglected. In contrast, weakly confined systems such as large CsPbBr_3_ QDs exhibit significant Coulomb correlations, necessitating a revised framework for biexciton radiative dynamics, one that has remained incomplete due to limited experimental insights. Here, we combine single‐particle photoluminescence (PL) spectroscopy with effective‐mass variational quantum Monte Carlo (VQMC) simulations to directly probe the biexciton geometry, exciton–phonon interactions, and radiative decay in CsPbBr_3_ QDs across a range of sizes and temperatures. We reveal that the biexciton‐to‐exciton radiative decay lifetime ratio is strongly modulated by inter‐exciton correlations, with a striking inversion of the expected decay lifetime hierarchy in the weak‐confinement regime. Specifically, at cryogenic temperatures, biexcitons exhibit anomalously longer radiative lifetime (148 ± 30 ps) than excitons (106 ± 16 ps), challenging conventional models. These findings uncover a correlation‐driven regime of multiexciton physics and suggest new routes for tailoring light–matter interactions for classical and quantum photonic applications.

## Graphical abstract

Single‐particle spectroscopy and variational quantum Monte Carlo simulations reveal that strong many‐body correlations fundamentally alter biexciton radiative recombination in weakly confined CsPbBr_3_ quantum dots. Intra‐ and inter‐exciton correlations reverse the conventional lifetime hierarchy, yielding biexcitons with longer radiative lifetimes than excitons at cryogenic temperatures. These findings uncover a correlation‐driven regime of multiexciton physics with important implications for quantum‐photonic technologies.

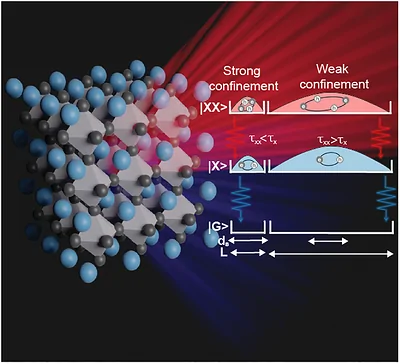

## 1 Main

In semiconductor quantum dots (QDs), excitons (X) and biexcitons (XX), composed of one and two electron‐hole (e‐h) pairs, respectively, are the elementary responses to optical excitations. The biexciton state |*XX*〉 decays to the ground state |*G*〉 through a |XX⟩→|X⟩→|G⟩ cascade, with the exciton state |*X*〉 as the intermediate state, yielding two temporally correlated photons, as illustrated in Figure 1a. Their recombination dynamics play a crucial role in the performance characteristics of QD‐based devices, such as lasing devices exploiting low‐threshold optical gain [1, 2] and quantum light sources capable of efficiently producing coherent single photons [3, 4, 5, 6] or correlated photon‐pairs [7, 8, 9, 10, 11]. Fundamentally, biexcitons represent a prototypical many‐body system, whose behavior is governed by the many‐body correlation among charge carriers, including the intra‐X and inter‐X correlations. These correlations not only profoundly influence device behaviors but also connect to fundamental aspects of quantum many‐body physics phenomena, such as collective excitations and responses, like superfluorescence [12, 13, 14] and Bose–Einstein condensation [15, 16].

**FIGURE 1:**
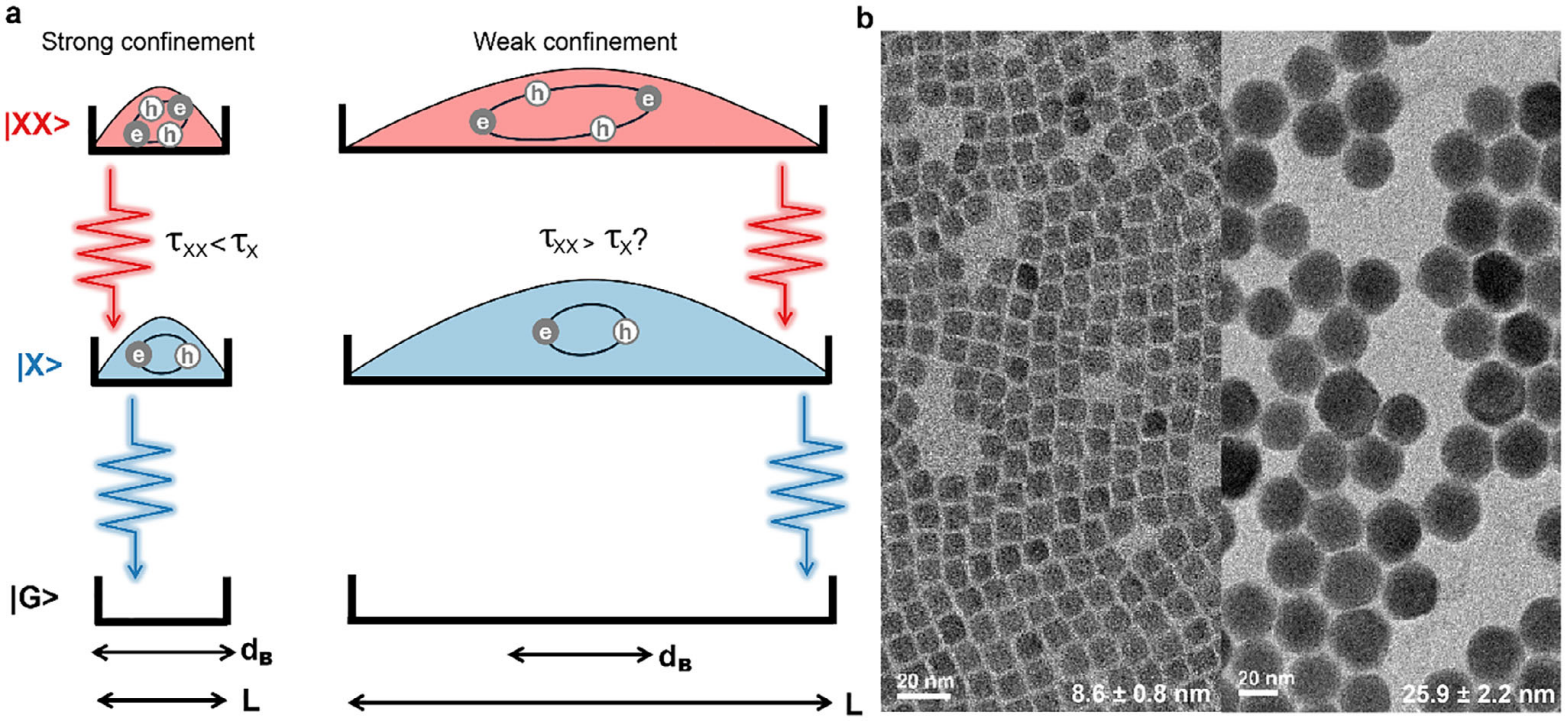
Biexciton emission cascade process in QDs. (a) In both strongly confined QDs (left column; with a QD size smaller than the exciton Bohr diameter d_B_) and weakly confined QDs (right column), biexcitons decay through a cascade process and generate two photons in sequence through the transition from |*XX*〉 to |*X*〉 (red upper arrows) and |*X*〉 to |*G*〉 (blue lower arrows). The transition energies, that is, the emitted photon energies, generally differ due to altered Coulomb interactions among the charge carriers. Moreover, decay lifetimes of the transitions in the cascade depend, next to combinatorial arguments, also on the inter‐X and intra‐X correlation, both of which scale with the QD size. (b) TEM images of the two CsPbBr_3_ QDs employed in this work, exhibiting mean edge lengths of 8.6 nm (left, intermediate‐confinement regime) and 25.9 nm (right, weak‐confinement regime), respectively.

So far, it is widely accepted that the biexciton exhibits a faster radiative decay than the exciton [17, 18, 19, 20, 21] (Figure 1a). Since each e‐h pair can recombine independently, the radiative lifetime of the biexciton is typically a quarter to half of the exciton, depending on the electronic structures and selection rules. However, as proposed by a theoretical study and very few experimental evidences [20, 21, 22, 23], this behavior may only hold in strongly confined systems, where inter‐X correlations play a secondary role. In contrast, in the weak confinement regime, the strong intra‐X correlation can give rise to a giant oscillator strength for the exciton recombination [20], enabled by the coherent delocalization of the exciton wavefunction across a large spatial volume [24]. Since the exciton state also serves as the final state of the biexciton decay, both intra‐X and inter‐X correlation may strongly influence the biexciton dynamics, potentially altering the relative lifetimes of the exciton and biexciton. However, an experimental validation of such unconventional behavior predicted by theoretical models is still lacking. For example, one very earlier work on CuCl single crystal reported very fast biexciton decay [17] compared to superradiant excitonic recombination. The lack of experimental observations can be attributed to two main reasons: on the one hand, there is very limited choice of highly tunable material systems allowing the engineering of many‐body correlations, often constrained by either the small available QD sizes (e.g., strongly confined system) or defects in the crystal structure which trap the exciton wavefunction (typically a practical concern for larger and more weakly confined systems); on the other hand, the biexciton recombination is typically also subject to efficient non‐radiative Auger‐Meitner recombination [25, 26], which further accelerates the biexciton non‐radiative decay. Yet, there are independent experimental and theoretical precedents which suggest that exciton and biexciton lifetimes can be comparable in systems dominated by many‐body correlations [21, 22, 23].

Among various QD systems, all‐inorganic lead‐halide perovskite QDs, that is, CsPbX_3_ (X = Cl, Br, and I), can be a suitable platform to explore the biexciton dynamics and inter‐X correlation. They are prepared via the well‐established colloidal syntheses [27, 28, 29], enabling precise control over their sizes with edge lengths ranging from 3 to 30 nm [28] and low size dispersion as well as excellent crystal quality and surface passivation (see the TEM images in Figure 1b). The synthesized QDs exhibit near‐unity photoluminescence (PL) quantum yield (QY) for both the exciton and the biexciton at low temperatures [3, 5, 6, 9]. The bright PL emission is attributed to the highly suppressed relaxation to the dark state at cryogenic temperatures and at zero magnetic field [11], in stark contrast to II‐VI and III‐V compounds [30]. Given the bright triplet character of the exciton in perovskite QDs, the radiative lifetime of the biexciton is expected to be one third of that of the exciton $\left(\tau_{XX}^{\mathrm{rad}}=\frac{\tau_{X}^{\mathrm{rad}}}{3}\;\right)$ in the strong confinement regime and neglecting Coulomb correlations (see Supporting Information Section II. G for details).

Moreover, recent studies have demonstrated that weakly confined CsPbBr_3_ QDs can generate highly coherent single photons [3, 4, 6] at cryogenic temperatures featuring over 50% Hong‐Ou‐Mandel (HOM) visibility [5] even without cavity integration. The key requirement for the realization of high indistinguishability is an optical coherence time approaching twice the radiative lifetime of the exciton. In CsPbBr_3_ QDs, this has been achieved predominantly through a record‐rapid radiative decay (∼100 ps), afforded by strong intra‐X correlation enabling single‐photon superradiance [6]. The involved coherent delocalization of the exciton wavefunction given by strong intra‐X correlation is also evident in single‐photon superabsorption [31], the time‐reversal counterpart to such fast emission process. Additionally, the biexciton binding energies (Δ_*XX*_) exceeding 14 meV indicate the presence of strong inter‐X correlation in such weakly confined QDs [32, 33, 34, 35]. The above‐mentioned unique properties candidate CsPbBr_3_ QDs as a suitable platform to systematically investigate many‐body correlations and the thus far poorly explored biexciton dynamics in semiconductor QDs in weak confinement regime.

In this work, we explored the biexciton dynamics in CsPbBr_3_ QDs (Figure 1b) of varying sizes and temperatures by combining single‐QD PL spectroscopy with effective‐mass variational quantum Monte‐Carlo (VQMC) simulations. We established a solid understanding of the biexciton geometry, XX‐phonon coupling, and radiative biexciton lifetime. Compared to the exciton, the biexciton exhibits a stronger coupling to phonons, which accounts for the 10% increase of the polaron size and enhances the biexciton binding energies. Contrary to the conventional understanding ($\tau_{XX}^{\mathrm{rad}}=\tau_{X}^{\mathrm{rad}}\;/3$), the radiative lifetime ratio of the biexciton to the exciton ($\tau_{XX}^{\mathrm{rad}}/\tau_{X}^{\mathrm{rad}}$) increases with increasing QD size or decreasing temperature, a behavior directly attributable to size‐ and temperature‐dependent inter‐X correlations. Remarkably, in weakly confined CsPbBr_3_ QDs at low temperatures, the biexciton exhibits a slower decay than the exciton, while both are entirely dominated by the radiative recombination. These findings offer a comprehensive framework for understanding the unconventional biexciton behavior in these highly engineerable colloidal CsPbBr_3_ QDs. It not only emphasizes the peculiar properties of the perovskite material but also represents a significant step toward the rational design of XX‐based photonic applications, for example, low‐threshold lasing and entangled‐photon generation.

## 2 Results and Discussions

To systematically explore the biexciton dynamics, we performed single‐QD PL spectroscopy experiments for QDs from five different synthesis batches with edge lengths ranging from 8.6 to 25.9 nm (for more information about the samples, see Figure S1 and Supporting Information Section I), thereby spanning from the intermediate to the weak confinement regime. At 4 K, individual QDs were excited with an average excitation density of ⟨N⟩∼0.4 (average exciton number generated per laser pulse) to resolve the biexciton emission. Two exemplary PL spectra from the 8.6 nm (upper panel) and 25.9 nm (lower panel) QD are plotted in Figure 2a, showing distinct emission peaks from the exciton, trion (X*), and biexciton (from high to low energies) [32, 33, 34, 35]. The spectrally integrated intensity increases linearly with excitation density (inset of Figure 2a), indicating the absence of excitation‐dependent non‐radiative channels, for example, Auger‐Meitner recombination, and similar QYs for all emitting species in these QDs. The absolute QY of the exciton in these particles has previously been reported to be near unity [3, 5, 6, 9, 36]. Hence, we conclude that the biexciton also possesses near‐unity QY.

**FIGURE 2:**
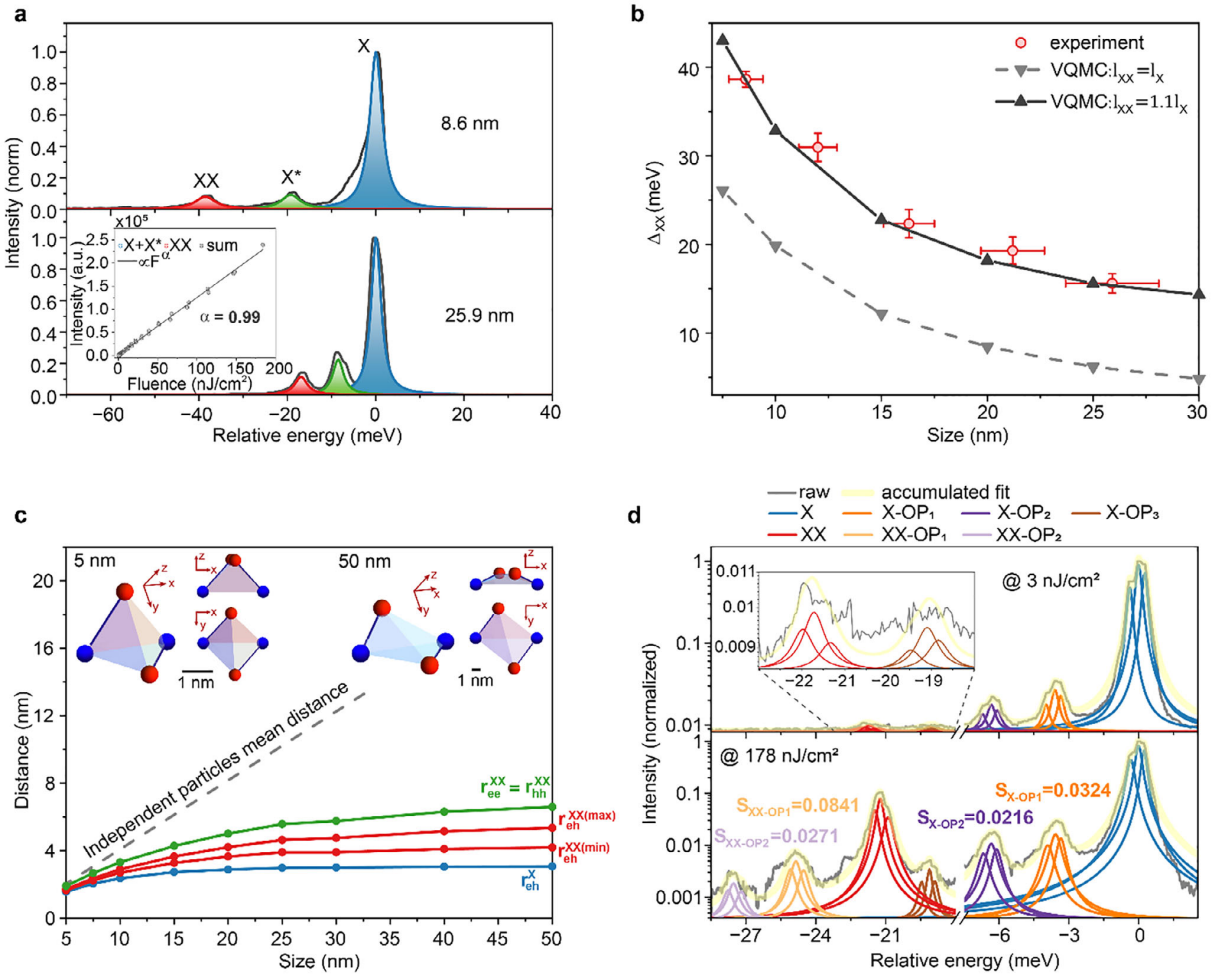
Photo‐physics of the biexciton in individual CsPbBr_3_ QDs at 4 K. (a) PL spectra of two individual QDs with an edge length of 8.6 nm (upper panel) and 25.9 nm (lower panel), respectively, at ⟨N⟩∼0.4 excitations per QD, with spectral fingerprints of the exciton (X), trion (X*), and biexciton (XX). The inset shows the integrated PL intensity, which h displays a linear behavior as a function of excitation fluence. (b) Size‐dependent biexciton binding energies (Δ_*XX*_). Experimental data are quantitatively reproduced by the VQMC calculations when considering a 10% larger polaron size in the biexciton regime. (c) Distance between the charge carriers and spatial geometry of the biexciton in different‐sized QDs, calculated from the most frequent charge distribution observed in VQMC simulations (blue dots: hole, red dots: electron). (d) X‐phonon coupling and XX‐phonon coupling in 16.3 nm QDs revealed via single‐QD spectroscopy. Two optical phonon modes at 3.5 meV (OP_1_) and 6.3 meV (OP_2_) of the XX are observed, with a stronger coupling to the biexciton compared to the exciton.

In Figure 2b, we consolidate our understanding of the Coulomb many‐body interaction among charge carriers by examining the size‐dependent biexciton binding energies (Δ_*XX*_) from both experimental spectra and the VQMC model. The latter, as an advanced theoretical model, is particularly well‐suited for studying strongly correlated systems due to its ability to provide highly accurate ground‐state wavefunctions while explicitly capturing electron correlation effects [20, 37, 38]. As indicated by the gray dashed line in Figure 2b, using the same parameters, for example, polaron size (*l_XX_*), effective mass, dielectric constant, in the biexciton regime as for the exciton clearly underestimates the surprisingly large Δ_*XX*_ found experimentally. Reproducing the experimentally found Δ_*XX*_ has previously been achieved by considering ad hoc a reduced effective dielectric constant (by around 5%–10% [33]) or a larger polaron size [37] in the biexciton regime. Here, we show that the VQMC model (details see Supporting Information Section II) displays excellent agreement with the experimental observations when considering a 10% larger biexciton polaron, that is,  *l_XX_* =  1.1*l_X_* (dark curve). Since a different polaron size for the exciton and biexciton may potentially indeed arise from the soft, ionic perovskite crystal, which responds differently to the different charge distributions [39], we will now assess in more detail first the biexciton geometry and then its coupling to lattice vibrations.

First, we analyze the size and spatial configuration of biexcitons in QDs of various sizes via VQMC simulations. Figure 2c shows that the biexciton adopts a distorted tetrahedron geometry, in contrast to the planar distorted square configuration in CdSe [20] or MAPbX_3_ [38] nanoplatelets. With increasing particle sizes, the biexciton tetrahedron is more distorted and asymmetric, and the overall spatial extent exceeds that of the exciton. Moreover, the e‐e ($r_{ee}^{XX}$) and h‐h ($r_{hh}^{XX}$) distances, which coincide due to similar electron and hole masses in CsPbBr_3_, are larger than the e‐h ($r_{eh}^{XX}$) distances, reflecting a net attractive Coulomb interaction for the biexciton. Furthermore, for QDs with sizes ≥ 10 nm, the calculated inter‐particle distances are much shorter than expected for non‐interacting carriers, evidence of a regime governed by interactions, rather than confinement.

Second, we assess the XX‐phonon coupling experimentally through the XX phonon replica in PL spectra of individual CsPbBr_3_ QDs at 4 K. For a representative QD (16.3 nm) with Δ_*XX*_ ≈ 22 meV, Figure 2d displays resolved spectral features of the exciton, biexciton, and their phonon replicas. A low laser fluence (3nJ/cm^2^) allows one to observe the zero‐phonon line of the exciton (blue curves) accompanied by three optical phonon replicas OP_1_ (orange curves), OP_2_ (purple curves), and OP_3_ (brown curves) [40], as well as the biexciton zero‐phonon line (red curves). All these spectral lines are present in triplicate at sub‐millielectronvolt spacings, reflecting the triplet exciton fine structure. Further details about the spectral fits are provided in Section III in Supporting Information. Resolving also the phonon replica of the biexciton requires excitation densities above 50nJ/cm^2^ (see Figure S3ab). The lower panel of Figure 2d displays the PL spectrum at a laser fluence of 178nJ/cm^2^, exhibiting two peaks besides the biexciton zero‐phonon line. These peaks are assigned to replicas of the optical phonon modes OP_1_ and OP_2_ due to identical energies observed for the exciton's OP_1_ and OP_2_ replica (Figure S3c).

Next, we quantify the phonon coupling strength (Huang‐Rhys factor, *S*) for both the exciton and biexciton by evaluating the intensity ratio of the phonon replica and the corresponding zero‐phonon line. For the OP_1_ and OP_2_ modes, *S* is 0.0324 and 0.0216 for the exciton and 0.0841 and 0.0271 for the biexciton, respectively. Measurements of several individual QDs consistently show an enhanced *S* for the biexciton (Figure S3d), particularly for the OP_1_ mode (Figure S3e). On average, the enhancement in coupling strength (*S_XX_*/*S_X_*) reaches a factor 2.75 and 1.60 for the OP_1_ and OP_2_ mode, respectively. Earlier studies on the single exciton [34, 40, 41, 42] have revealed that the exciton‐phonon coupling strength *S* of the exciton increases with decreasing QD size, in part due to enhanced atomic mobility at the QD surface [41]. This enhanced coupling in smaller QDs is especially pronounced for the two lower‐energy phonon modes OP_1_ and OP_2_ originating from the octahedral tilting. Such size‐dependent trends for the exciton are overall consistent with the stronger polaronic coupling of the biexciton with a larger spatial geometry compared to the exciton, which enhances the Coulomb interaction and thereby increases Δ_*XX*_, as shown in Figure 2b. In this regard, our experimental observation of stronger XX–phonon coupling, together with the developed VQMC method, resolves the previously mentioned discrepancy between measured and predicted Δ_*XX*_ if phonons are not accounted for.

We subsequently proceed to the main topic of this study: the dynamical behavior of both the exciton and biexciton at the single‐particle level. For each of the studied individual QDs, spectrally resolved PL lifetimes were measured at the exciton and biexciton peak energies. Figure 3a presents the typical time‐resolved PL (TRPL) traces of the exciton (blue curve) and biexciton (red curve) from an 8.6 nm (left panel) and 25.9 nm (right panel) QD, respectively. Lifetimes are provided as the duration of a PL intensity decrease to 1/*e* of its value right after photoexcitation (see details regarding the data analysis in the Supporting Information). In the 8.6 nm QD, with a size just above the Bohr diameter, the biexciton exhibits a smaller lifetime (351 ps) than the exciton (490 ps). Such a relation, with a biexciton decaying faster than the exciton, represents the conventional case in semiconductors. However, this faster biexciton decay represents a small deviation from the expected ratio $\tau_{XX}^{\mathrm{rad}}=\tau_{X}^{\mathrm{rad}}/3$ and may already foreshadow the onset of correlation effects, consistent with the intermediate‐confinement regime applicable for this QD. Strikingly, however, the weakly confined 25.9 nm QD shows a contrasting behavior: the biexciton exhibits a much *longer* lifetime (208 ps) than the exciton (108 ps). To systematically investigate this unconventional trend, we measured over 10 individual QDs from each of the five synthesis batches differing in average QD size (see Figure S1). The left panel of Figure 3b summarizes the mean decay lifetime of the exciton and the biexciton for these batches as a function of their edge lengths. The error bars (representing standard deviations) report the QD‐to‐QD variations within the sample (see Figure S4 for the distribution of lifetimes of individual QDs). For both the exciton and biexciton, the radiative decay lifetime gets faster with QD sizes. However, while the exciton lifetime decreases strongly from 414 to 106 ps for QD sizes from 8.9 to 25.9 nm, respectively, the biexciton lifetime decreases more moderately, from 304 to 148 ps. Both the individual size‐dependent trends and the relative behavior of exciton and biexciton are in stark contrast to the room‐temperature observations [43, 44]. Additionally, we performed time‐resolved PL measurements, at 4 K, on an ensemble film of the 25.9 nm quantum‐dot batch using a streak camera. The independent ensemble measurements yield same results, providing additional experimental evidence that fully supports the single‐QD measurements (see Figure S7). We previously attributed the size‐dependent trend of the exciton lifetime to single‐photon superradiance, a giant‐oscillator‐strength phenomenon arising from the wavefunction delocalization throughout the entire QD volume [6]. For the exciton case, the |*X*〉 state is a correlated state, while the ground state is an uncorrelated empty state, enabling a size‐dependent acceleration of the radiative lifetime [45]. In the case of the biexciton, however, both the initial |*XX*〉 and final |*X*〉 state are correlated states, leading to a weaker acceleration of the decay lifetime and rendering the biexciton decay slower than in the exciton for QDs larger than 17 nm (Figure 3b).

**FIGURE 3:**
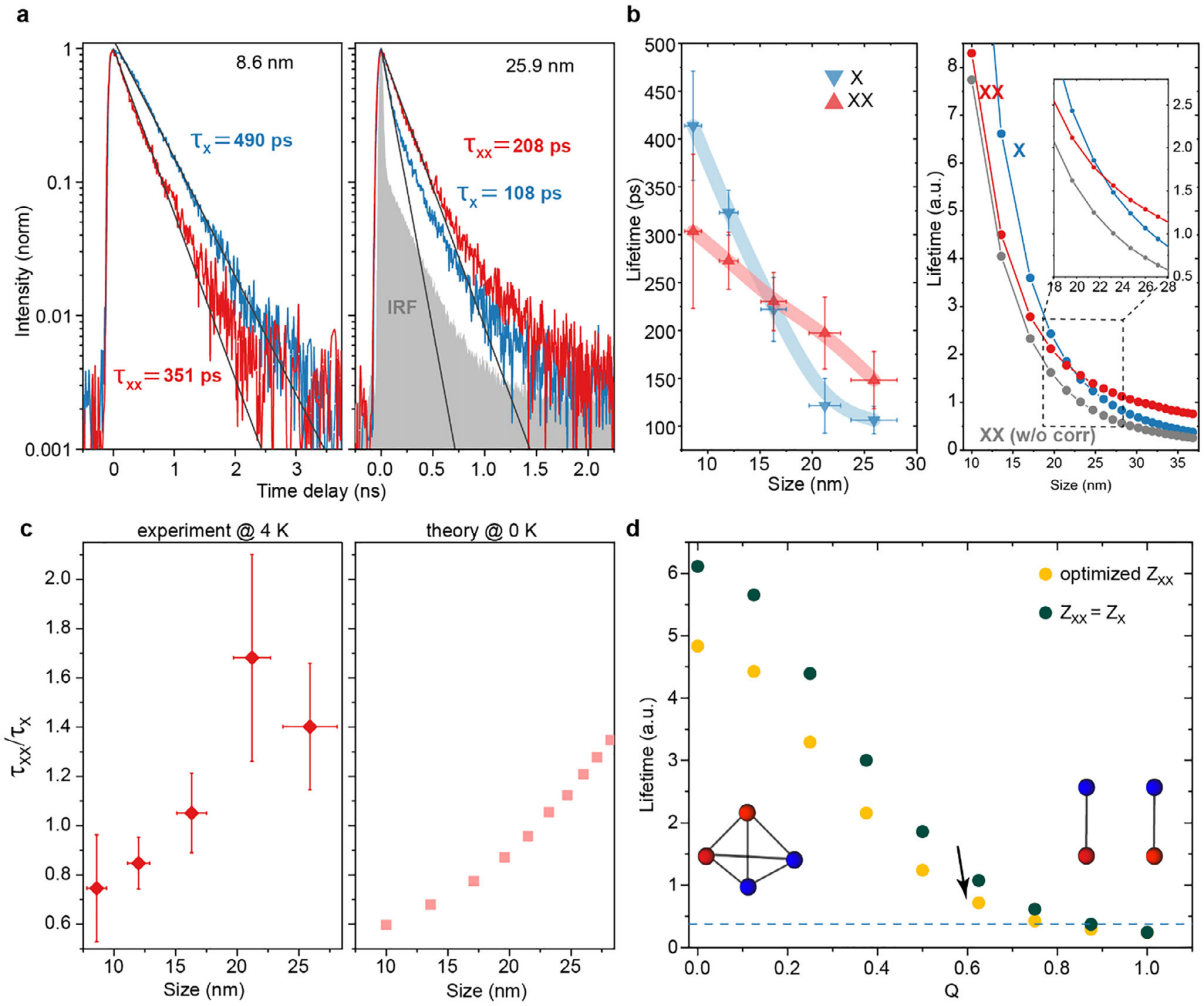
Size‐dependent biexciton decay lifetime at 4 K. (a) Exemplary TRPL traces of exciton (blue) and biexciton (red) in an individual 8.6 nm (left) and 25.9 nm (right) CsPbBr_3_ QD. The instrument response function (IRF) is shown as the gray area, and the mono‐exponential fits over the selected time windows are indicated in dark. (b) Left panel: Mean lifetime of the exciton (blue markers) and biexciton (red markers) as a function of the mean edge length of each sample, obtained through measurement of 10 individual QDs per sample. Error bars represent the standard deviation. Shaded areas serve as guides for the eyes. Right panel: Calculated decay lifetime of the exciton (blue) and biexciton with (red) or without (gray) inter‐X correlation, as a function of the QD size. The inset shows a zoom‐in of the cross‐over region. (c) Mean lifetime ratio between biexciton and exciton as a function of the mean QD edge length of each sample. (d) Calculated decay lifetime of biexciton in a 36.8 nm QD as a function of the X‐X dissociation parameter, Q, for optimized *Z_XX_* (yellow), or fixed *Z_XX_* = *Z_X_*  (dark green). The arrow points at the actual biexciton ground state. For comparison, the dashed line shows the exciton decay lifetime. The sketches represent the limits of fully bound and dissociated biexciton.

To find the origin of the unconventional biexciton dynamics, we calculate the radiative lifetime of the exciton and biexciton in cuboidal QDs at 0 K based on the VQMC model. As plotted in the right panel of Figure 3b, both the exciton (blue markers) and the biexciton (red and gray markers) exhibit shorter radiative lifetime for increasing QD size, indicating an enhanced oscillator strength due to increasing intra‐X correlation in larger QDs. In the absence of inter‐X correlation, the biexciton lifetime (gray markers) is a lower bound of the exciton's, regardless of the QD size, consistent with the conventional understanding of the biexciton dynamics. However, when the inter‐X correlation is accounted for in the VQMC model (red markers), the size‐dependent acceleration of the biexciton lifetime, while still present, drastically diminishes, especially in the weakly confined regime. As a result, the biexciton lifetime becomes *longer* than the exciton in larger QDs, which aligns well with the experimental observations. To provide a direct and quantitative comparison of the intrinsic difference between exciton and biexciton dynamics, we now calculate the lifetime ratio between biexciton and exciton ($\frac{\tau_{XX}}{\tau_{X}}$) for each individual QD and present the statistics (average value and error bar) of this ratio for each batch in Figure 3c. This representation clearly reveals the size‐dependent trend and enables a precise comparison between experiment and theory from the intermediate‐ to the weak‐confinement regime. Notably, experiment and theory are in excellent agreement and clearly reveal the transition from conventional ($\frac{\tau_{XX}}{\tau_{X}}<1$) to unconventional ($\frac{\tau_{XX}}{\tau_{X}}>1$) biexciton dynamics. This result is not a consequence of the polaronic nature of halide perovskites (see Section IV A in Supporting Information). The pivotal role of inter‐X correlation in determining the biexciton lifetime is surprising despite the only small involved energy (a few milli‐electronvolts) [37]. Unlike in other materials, the unconventional biexciton behavior emerges in weakly confined CsPbBr_3_ QDs due to their low structural disorder at cryogenic temperatures and high PLQY, which together enable strong inter‐X correlations.

Closer insight into the physical mechanisms contributing to the radiative decay of exciton and biexciton can be obtained by inspecting their wavefunctions, which determine the matrix dipole element (see Section II in the Supporting Information for details). These wavefunctions can be written as Ψ_*X*_ = Φ_*X*_ *F_X_* and Ψ_*XX*_ = Φ_*XX*_ *F_XX_*. Φ is the function of non‐interacting electrons and holes, while *F* is the electronic correlation factor. The former is often used to explain radiative rates in strongly confined QDs [46, 47, 48]. However, concepts such as the electron‐hole overlap fail to predict rates in weakly confined QDs. Evidence of this is that the mean electron‐hole distances (Figure 2c) remain constant for lateral sizes beyond 20 nm, and yet the radiative lifetime continues to decrease (Figure 3b,c). In superradiant QDs, it is the correlation effects (present in *F*), that is, the inter‐dependence of the motion of two or more particles, that matter [6, 31]. For excitons, the (hydrogen‐like) Slater factor reads as $F_{X}=e^{-Z_{X}\;r_{eh}}$. Its radiative rate is proportional to *Z_X_*, a parameter denoting the strength of the electron‐hole correlation [45]. For biexcitons, an enhanced Hylleraas function is used:

(1) $$F_{XX}=e^{-Z_{XX}\left(\frac{s}{2}\right)}\;\cosh\left(Z_{XX}Q\frac{t}{2}\right)e^{Z_{XX}\frac{\beta r_{12}}{1+Z_{XX}\alpha r_{12}}}e^{Z_{XX}\frac{\beta r_{ab}}{1+Z_{XX}\alpha r_{ab}}}$$

The variational parameters in *F_XX_* account for different many‐body effects: *Z_XX_* describes the electron‐hole correlation, α and β the electron‐electron and hole‐hole correlation, and *Q* the degree of dissociation of biexciton (specific to the inter‐X correlation). Our simulations show that α and β play a marginal role in the biexciton dynamics (Figure S8). By contrast, Figure 3d shows that *Q* induces the reversal of X‐XX decay lifetimes. For a QD of lateral size 36.8 nm, perfectly bound biexcitons (*Q*  =  0) decay slower than the exciton (horizontal line), while dissociated ones (*Q*  =  1) do so faster. This is true regardless of the specific choice of *Z_XX_* (green or yellow markers). The variationally optimized (lowest‐energy) biexciton shows up at *Q* ≈ 0.6 (see arrow). Thus, the partial X‐X association makes the biexciton ground state decay slower than the exciton. *Q* can be directly related to the Slater intra‐X and inter‐X correlation factors as *Z* ^intra^ = *Z_XX_* (1 + *Q*)/2 and *Z* ^inter^ = *Z_XX_* (1 − *Q*)/2 (see Section IVB in the Supporting Information). Only the latter is proportional to the radiative rates of the biexciton (Figure S9). This implies that the unconventional decay dynamics of the biexciton cannot be explained by looking at the individual X (*Z* ^intra^). It is a genuine four‐body effect involving the inter‐X correlation (*Z* ^inter^). In short, the radiative lifetime depends not on the electron‐hole overlap, but on that between exciton and biexciton. When *Q*  =  1 (*Z* ^inter^ =  0), the biexciton behaves as two uncorrelated excitons and the overlap is large. However, when *Q*  =  0 (*Z* ^inter^ = *Z* ^intra^  = *Z_XX_* /2), the biexciton behaves as a truly distinct particle, with different charge distribution and fermionic statistics. This reduces the overlap and, thus, the radiative rate.

In addition to the QD size, temperature also impacts the Coulomb correlations. Thermal energy can induce a stronger dynamic structural disorder in the crystal, which in turn suppresses both the intra‐ and inter‐X correlation as temperature increases. This trend has been supported by earlier studies, which unveiled longer lifetime of the exciton at elevated temperatures [6] and significantly reduced Δ_*XX*_ [49] at room temperature. Consequently, the corresponding exciton and biexciton lifetime may shift to the conventional regime as temperature increases. To explore this scenario, we study the emission dynamics of the exciton and biexciton in large 25.9 nm QDs at higher temperatures. At 50 K, we could still spectrally resolve the emission of exciton, trion, and biexciton, as displayed in Figure 4a. Like the above‐discussed low‐temperature measurements (inset of Figure 4a), a linear increase of their emission intensities with increasing excitation density suggests almost identical PLQYs for all the emitting species. TRPL traces at 50 K, presented in Figure 4b, reveal that both exciton and biexciton decay more slowly than at 4 K, and the exciton again decays more slowly than the biexciton. On average, the lifetime of the exciton and biexciton are increased to 461 and 308 ps, respectively. These findings confirm that increased thermal disorder weakens the inter‐X correlations, restoring the traditional decay dynamics of the exciton and biexciton.

**FIGURE 4:**
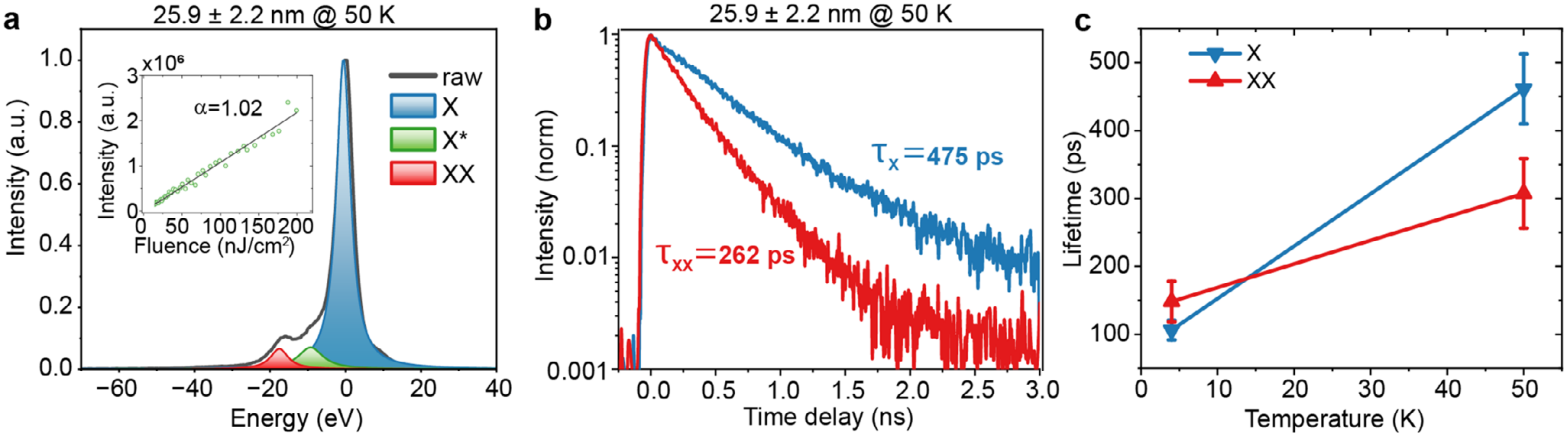
Temperature‐dependent biexciton lifetime. (a) Representative PL spectrum of a 25.9 nm CsPbBr_3_ QD at 50 K with a spectrally resolved emission of X (blue), X* (green), and XX (red). Inset: Excitation‐dependent PL intensity; a linear increase and an earlier revealed near‐unity PLQY of the exciton [6] suggest the near‐unity PLQY of all the emissive species. (b) Exemplary TRPL traces of the exciton (blue curve) and biexciton (red curve). (c) Temperature‐dependent lifetime of the exciton (blue markers) and biexciton (red markers). Error bars represent the standard deviation.

## 3 Conclusion

In this work, we uncover that biexcitons in large and weakly confined CsPbBr_3_ perovskite QDs at liquid‐helium temperatures radiate *more slowly* than the exciton, in stark contrast to the conventional models for excitons and biexcitons, which predicts the inverse relationship, based on the approximation of independent electrons and holes. While such conventional model closely describes the case of strongly confined electronic systems and has, therefore, been successful in explaining decay lifetimes in a broad range of organic molecules and semiconductor nanostructures, we here introduce, probe, and explain the emerging case of a weakly confined emissive semiconductor such as large individual CsPbBr_3_ QDs. Combining single‐QD PL spectroscopy and VQMC simulations, we attribute the atypical size‐dependent trends of the exciton and biexciton radiative lifetimes at liquid‐helium temperature to the strong many‐body correlations both within and between excitonic states. These intra‐ and inter‐X correlations can be systematically tuned by QD size and crystal temperature. Furthermore, we propose that the size‐dependent biexciton binding energy and radiative lifetime can be quantitatively reproduced by accounting for the size and (asymmetric tetrahedron) shape of the biexciton state, which is characterized by both strong electronic correlation and strong coupling to phonons. This systematic experimental and theoretical study of highly engineerable lead‐halide perovskite QDs in the hitherto poorly explored weak confinement regime with strong‐correlation supports future efforts to realize advanced classical and quantum photonic technologies that exploit biexciton dynamics, such as low‐threshold lasing and entangled‐photon generation.

## 4 Methods

### 4.1 Synthesis

The studied CsPbBr_3_ QDs are synthesized following the method reported in ref. [28, 29], with some modifications.

More details about the samples are provided in the Supporting Information.

### 4.2 Sample Preparation

The preparation consists of two steps: dilution and spin‐coating. For QDs with sizes of 8.6–12.0 nm, dispersed in cyclohexane, the QD dispersion with a concentration of ∼1 mg/ml is diluted by a factor of 100 in cyclohexane (ACROS, 99.85% extra dry over molecular sieves). The solution is further diluted by another factor of 100 in a 3‐mass% solution of SEBS in cyclohexane. Fifty microliters of the final solution are spin‐coated onto an intrinsic crystalline Si wafer with a 3‐µm‐thick thermal‐oxide layer at 50 r.p.s for 60 s. For QDs with a size of 16.3–25.9 nm, dispersed in toluene, the QD dispersion with a concentration of ∼1 mg/ml is diluted by a factor of 80 in toluene (ACROS, 99.85% extra dry over molecular sieves). The solution is further diluted by another factor of 100 in a 3‐mass% solution of polystyrene (ALDRICH, average Mw ∼ 280 000) in toluene. 50 µL of the final solution are spin‐coated onto an intrinsic crystalline Si wafer with a 3‐µm‐thick thermal‐oxide layer at 50 r.p.s for 60 s.

### 4.3 Optical Characterization

For single‐particle spectroscopy, a home‐built µ‐PL setup is used. The samples were mounted on xyz nano‐positioning stages inside an evacuated liquid‐helium closed‐loop cryostat (MONTANA INSTRUMENTS) and cooled down to a targeted temperature of 4 K. Single QDs were excited by means of a fiber‐coupled excitation laser at an energy of 2.585 eV (480 nm) with a repetition rate of 80 MHz (TOPTICA, < 200 fs pulses), which is focused (1/*e* ^2^ diameter = 2.4 µm) on the sample by a microscope objective (NA = 0.8, 100×). The emitted light is collected by the same objective and passed through a 90:10 beam splitter and a long‐pass filter at 500 nm. A monochromator coupled to a back‐illuminated CCD (Princeton Instruments) is used for spectra measurements. Spectra are measured with a grating of 300 (or 1800) lines/mm, blaze 500 nm, yielding around 1 (or 0.25) meV spectral resolution. An APD and a TCSPC Module (PicoQuant, PicoHarp) are used for the lifetime measurement.

### 4.4 Variational Quantum Monte‐Carlo Simulations

A detailed description of the model and computational aspects can be found in refs. [37, 38, 50, 51, 52] and Supporting Information.

## Author Contributions

**Jose L. Movilla**: formal analysis, data curation. **Juan I. Climente**: supervision, data curation, formal analysis, writing – review and editing, visualization, funding acquisition. **Chenglian Zhu**: investigation, formal analysis, data curation, writing – original draft. **Ihor Cherniukh**: data curation, investigation. **Petra Hoffmann**: investigation, data curation. **Simon C. Boehme**: writing – review and editing, data curation, formal analysis. **Maksym V. Kovalenko**: visualization, writing – review and editing, formal analysis, data curation, supervision, funding acquisition. **Willem P. A. Verheijen**: validation, data curation. **Maryna I. Bodnarchuk**: supervision, funding acquisition, data curation. **Leon G. Feld**: investigation, data curation. **Josep Planelles**: supervision, data curation. **Gabriele Rainò**: funding acquisition, visualization, writing – review and editing, formal analysis, data curation, supervision.

## Conflicts of Interest

The authors declare no conflicts of interest.

## Supporting information




**Supporting File**: adma74849‐sup‐0001‐SuppMat.pdf.

## Data Availability

The datasets generated during and/or analyzed during the current study are available in a repository, https://doi.org/10.3929/ethz‐c‐000803926.
