# Supplementary material for: Unconventional Biexciton Dynamics in Superradiant Perovskite Quantum Dots Driven by Many‐Body Correlations
Source: Adv Mater. 2026 Aug 31;38(59):e74849. doi: 10.1002/adma.74849 (PMC13689886; doi:10.1002/adma.74849)
Supplement: Supplementary file 1 [file ADMA-38-e74849-s001.pdf]

Supplementary Information

Unconventional Biexciton Dynamics in Superradiant Perovskite Quantum Dots Driven by Many-Body Correlations

Chenglian Zhu,[1,2,€] *Jose L. Movilla*[3,€], *Petra Hoffmann,*[1,2] *Ihor Cherniukh,*[1,2] *Willem P. A. Verheijen,*[1,2] *Leon G. Feld,*[1,2] *Josep Planelles,*[4] *Maryna I. Bodnarchuk,*[1,2] *Simon C. Boehme,*[1,2] *Juan I. Climente,*[4*] *Maksym V. Kovalenko*[1,2*] *and Gabriele Rainò*[1,2*]

[1] Institute of Inorganic Chemistry, Department of Chemistry and Applied Biosciences, ETH Zürich, CH-8093 Zürich, Switzerland

[2] Laboratory for Thin Films and Photovoltaics, Empa – Swiss Federal Laboratories for Materials Science and Technology, CH-8600 Dübendorf, Switzerland

[3] Departament d'Educacio i Didactiques Especifiques, Universitat Jaume I, 12080 Castello, Spain

[4] Departament de Química Física i Analítica, Universitat Jaume I, 12080 Castelló, Spain

Emails: climente@uji.es, rainog@ethz.ch, mvkovalenko@ethz.ch

[€]These authors equally contribute to this work.

Table of Contents

I. Sample synthesis ... 3

A. Synthesis of $CsPbBr_3$ QDs ... 3

II. Quantum Monte-Carlo simulations ... 5

A. X and XX Hamiltonians ... 5

B. Variational wave functions ... 8

C. Integration method ... 9

D. Biexciton geometry ... 10

E. Radiative rates ... 11

F. Material Parameters for $CsPbBr_3$ ... 12

G. Ratio between exciton and biexciton recombination rates in the strong confinement limit ... 12

III. Further experimental details ... 16

IV. Further theoretical results ... 21

A. Robustness against polaronic model and parameters ... 21

B. Influence of different types of correlation ... 22

REFERENCES ... 25

I. Sample synthesis

The synthesis of $CsPbBr_3$ quantum dots (QDs) was conducted according to the ref.[1] with small modifications, as described below.

A. Synthesis of $CsPbBr_3$ QDs

Materials

Lead bromide ($PbBr_2$, 99.999%, Sigma Aldrich), cesium carbonate (Cs_2CO_3, 99.9%, Sigma Aldrich), trioctylphosphine oxide (TOPO, min. 90%, Strem Chemicals), bis(2,4,4-trimethylpentyl)phosphinic acid (TMPPA, 90%, Fluorochem), oleic acid (OA, 90%, Sigma Aldrich), phosphorus(V) oxychloride (99%, Sigma Aldrich), 2-aminoethan-1-ol (⩾99.0%, Sigma Aldrich), acetic acid (>99.8%, Sigma Aldrich), triethylamine (99%, Sigma Aldrich), 2-octyl-1-dodecanol (97%, Sigma Aldrich), sorbitan trioleate (Span® 85, Fluka), lecithin (⩾97%, Roth), acetone (≥99.5%, Sigma Aldrich), mesitylene (99%, Thermo Scientific Chemicals), toluene (99.9%, Sigma-Aldrich), n-octane (min. 99%, Carl Roth), cychohexane (HPLC grade, Aldrich).

QD synthesis

$PbBr_2$-TOPO stock solution for 8.6 and 12.0 nm $CsPbBr_3$ QDs was prepared by dissolving 367 mg of $PbBr_2$ and 2.15 g of TOPO into 5 ml of n-octane at 120 °C, followed by cooling down and dilution with 20 ml of hexane. Cs-TMPPA stock solution for 8.6 and 12.0 nm QDs was prepared by reacting 100 mg of Cs_2CO_3 with 1 ml of TMPPA in 2 ml of n-octane at 100 °C, followed by cooling down and dilution with 27 ml of hexane. $PbBr_2$-TOPO stock solution for 16.3 and 25.9 nm QDs was prepared by dissolving 367 mg of $PbBr_2$ and 2.15 g of TOPO into 5 ml of mesitylene at 120 °C, followed by cooling down and dilution with 20 ml of mesitylene. Cs-OA stock solution for 16.3 and 25.9 nm QDs was prepared by reacting 100 mg of Cs_2CO_3 with 1 ml of OA in 2 ml of mesitylene at 100 °C, followed by cooling down and dilution with 27 ml of mesitylene.

2-octyl-1-dodecylphosphoethanolamine (PEA-C8C12) and 2-ammonioethyl sorbitan trioleate (Span 85®) phosphate (PEA-sorbitan-trioleate) ligands were synthesized following the protocol by Morad et al.[2] and dissolved in mesitylene and toluene, respectively, to form 0.1

M solutions. For the synthesis of PEA-sorbitan-trioleate, solution of sorbitan trioleate (5.98 g, 6.25 mmol; 1 eq.) dissolved in dry THF (6.25 ml) along with triethylamine (0.957 ml, 6.875 mmol; 1.1 eq.) was added dropwise under vigorous stirring to a solution of phosphorous oxychloride (0.695 ml, 7.5 mmol; 1.2 eq.) in THF (1 ml) on an ice water bath. The mixture was subsequently kept at 20 °C for 18 minutes to complete the reaction. Next, ethanolamine (0.452 ml, 7.5 mmol; 1.2 eq.) and triethylamine (2.09 ml, 15 mmol; 2.4 eq.) in THF (9.375 ml) were added dropwise under vigorous stirring to the reaction mixture kept in room-temperature water bath. Subsequently, the mixture was heated to 40 °C for 20 min to complete the ring closure. The reaction mixture was filtered to remove precipitated triethylamine hydrochloride and the filtrate solution was rotary evaporated to obtain a yellowish viscous substance. The remains were dispersed in diethyl ether (15 ml), kept in freezer for 1 hour and filtered again to remove the remaining triethylamine hydrochloride. The solution was rotary evaporated again and the purification process with dissolution in diethyl ether (40 ml), freezing and filtration was repeated twice. A viscous residue obtained after rotary evaporation was dissolved in THF (2 ml) and a mixture of acetic acid (1.43 ml) and distilled water (0.642 ml) was added at 70 °C. After 33 min at this temperature, ring scission at the P-N bond was complete and the product was separated by beating with ~40 ml of cold acetone. After keeping the mixture for 2 hours in freezer, the supernatant was discarded and jelly precipitate was washed one more time with cold acetone. The precipitate was purified again by dissolving it in diethyl ether, filtering out the remaining triethylamine hydrochloride, and removing the solvent by rotary evaporation. The pinkish product was dried at 40 °C under vacuum overnight.

8.6 and 12.0 nm $CsPbBr_3$ QDs were synthesized according to Akkerman et al.[1] with slight modifications. For the synthesis of 8.6 nm $CsPbBr_3$ QDs, 0.8 ml of Cs-TMPPA stock solution were injected into a mixture of 3.5 ml of hexane and 1.6 ml of $PbBr_2$-TOPO stock solution under vigorous stirring at room temperature. After 3 min, 130 µl of 0.1 M PEA-C8C12 in mesitylene were injected and the crude solution was stirred for an additional 2 min, followed by rotary evaporation until 2 ml remained. Next, the QDs were destabilized with 1 ml of acetone, precipitated by centrifugation at 10000 rpm for 1 min, and redispersed in 1 ml of cyclohexane. The purification was repeated one more time using 0.5 ml of acetone and the QDs were redispersed in 1 ml of cyclohexane and 20 µl of 0.01 M PEA-C8C12. For the synthesis of 12.0 nm $CsPbBr_3$ QDs, 1.5 ml of Cs-TMPPA stock solution were injected into a mixture of 1.75 ml of hexane and 3 ml of $PbBr_2$-TOPO stock solution under vigorous stirring at room

temperature. After 4 min, 0.13 ml of 0.1 M PEA-C8C12 in mesitylene were injected into a 3 ml part of the crude solution, followed by stirring for an additional 2 min and rotary evaporation until 2 ml remained. The purification was carried out in the same way as for the 8.6 nm QDs. 16.3 nm, 21.2 nm and 25.9 nm $CsPbBr_3$ QDs were synthesized by adapting the $PbBr_2$-TOPO approach by Akkerman et al. [1] with a slow injection of Cs-OA and $PbBr_2$-TOPO precursors at higher temperature. The 16.3 nm and 21.2 QDs were capped with 2-Ammonioethyl sorbitan trioleate (Span 85®) phosphate ligand, precipitated twice with acetone and redispersed in toluene. The 25.9 nm QDs were capped with lecithin, washed once with an ethyl acetate-acetone mixture (2:1, v/v) and once with ethanol, and then redispersed in toluene.

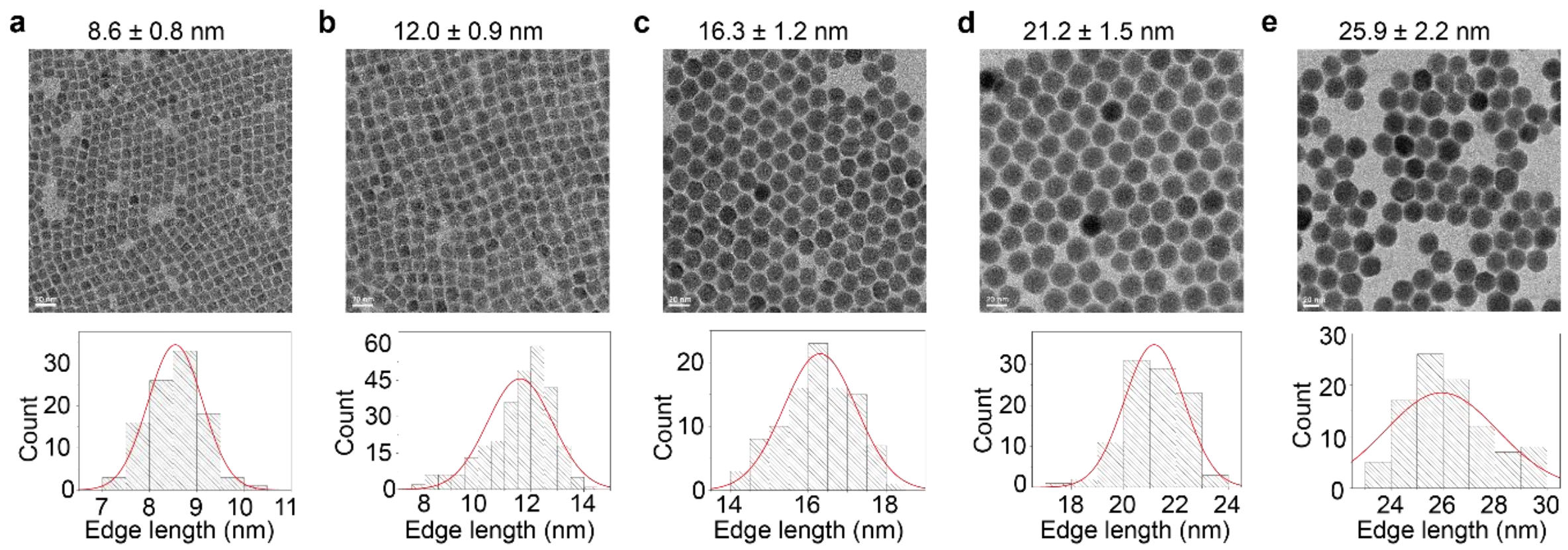

Fig. S1| Size characterization of the studied samples. A-e, The TEM images and corresponding size distribution of the samples with mean edge lengths of ca. 8.6 nm, 12.0 nm, 16.3 nm, 21.2 nm, and 25.9 nm, respectively.

II. Quantum Monte-Carlo simulations

A. X and XX Hamiltonians

We calculate exciton (X) and biexciton (XX) ground state energies and wave functions from $k \cdot p$ Hamiltonians for two uncoupled (conduction and valence) bands. These account for 3D (cubic-shaped) spatial confining potentials, dielectric confinement, and long-range[3] and short-range[4] interactions between carriers based on a Bajaj potential[5], a Haken-like model of polaronic interactions[6].

The exciton and biexciton Hamiltonians read, in atomic units (a.u.):

$$H_X = \sum_{i=e,h} \left(\frac{\hat{\mathbf{p}}_i^2}{2m_i} + V_i \right) + V_c(\mathbf{r}_e, \mathbf{r}_h), \qquad (S1)$$

$$\mathrm{H_{XX}} = \sum_{i=e_1,e_2,h_a,h_b} \left(\frac{\hat{\mathbf{p}}_i^2}{2m_i} + V_i \right) + V_c(\mathbf{r}_{e_1}, \mathbf{r}_{e_2}) + V_c(\mathbf{r}_{h_a}, \mathbf{r}_{h_b}) + \sum_{i=e_1,e_2} \sum_{j=h_a,h_b} \mathrm{V_c}(\mathbf{r}_i, \mathbf{r}_j), \qquad (S2)$$

where m is the effective mass, and $\hat{p}$ the momentum operator. The single particle potential is $V_i = V_i = V_i^{conf} + V_i^{self}$, where V_i^{conf} is the 3D confining potential. In our model, we describe the QDs as cuboids with lateral dimension L. For holes, we take $V_h^{conf} = 0$ inside the cuboid and infinite outside. For electrons, $V_e^{conf} = E_{gap}$ (the bulk band gap) inside the cuboid and infinite outside it. V_i^{self} is the self-energy potential. $V_c(\mathbf{r}_i, \mathbf{r}_j)$ terms represent the Coulomb interaction between carriers. Both V_i^{self} and $V_c(\mathbf{r}_i, \mathbf{r}_j)$ account for dielectric confinement by using quantum box image charges, with inclusion of long- and short-range interactions by means of a Bajaj potential.

In bulk systems, the Bajaj potential reads as follows (in a.u.)[5]:

$$V_c^{bulk}(\mathbf{r}_i, \mathbf{r}_j) = \frac{q_i q_j}{\varepsilon_s\, r} + \left(\frac{\varepsilon_\infty}{\varepsilon_s}\right)^{\gamma} \frac{q_i q_j}{\varepsilon^*\, r} \frac{e^{-\beta_i r} + e^{-\beta_j r}}{2}. \qquad (S3)$$

Here, r is the distance between charges i and j, q the elementary charge (positive or negative) and ε_s the static dielectric constant. The term $\left(\frac{1}{\varepsilon_\infty} - \frac{1}{\varepsilon_s}\right) = \frac{1}{\varepsilon^*}$ represents the ionic screening of carriers, with ε_∞ the high frequency dielectric constant and $\beta_i = 1/l_i$, with l_i the polaron radius of carrier i. The latter is defined as:

$$l_i = \sqrt{\frac{\hbar^2}{2\, m_i^b\, E_{LO}}}, \qquad (S4)$$

with E_{LO} the LO phonon energy and m_i^b the bare effective mass, which relates to the polaronic one as $m_i = m_i^b\,(1 + \alpha/6)$, α being the Fröhlich coupling constant. Note that the first term in Eq. $S3$ is a Coulomb interaction with full dielectric screening (electronic plus ionic, ε_s), which prevails at long distances, $r \gg l_i$. On the other hand, the second term is a short-range (Yukawa) one, which starts prevailing at distances $\mathrm{r} \lesssim l_i$, where the ionic contribution to

dielectric screening is lost (ε_∞ dominates). The term $(\varepsilon_\infty/\varepsilon_s)^\gamma$, with γ a material dependent parameter, is the distinct trait of Bajaj potential as compared to the original Haken one. The goal of this term is to improve (phenomenologically) the estimates of exciton binding energies[5].

In QDs, the potential Eq. S3 is renormalized due to the dielectric confinement. In metal halide perovskite QDs, $V_c(\mathbf{r}_i,\mathbf{r}_j)$ (considering dielectric confinement with interparticle distance-dependent polaronic contributions) can be obtained by slightly modifying the image charge-based expressions provided in ref.[3] for rigid semiconductor cuboidal structures. In a.u., the modified potential is expressed as follows[4]:

$$V_c(\mathbf{r}_i,\mathbf{r}_j) = \sum_{k=C,Y} \sum_{l=-\infty}^{\infty} \sum_{m=-\infty}^{\infty} \sum_{n=-\infty}^{\infty} \frac{q_i q_j \, q_{lmn,k} \, f_{lmn,k}(\mathbf{r}_i,\mathbf{r}_j)}{r_{lmn}}. \quad (S5)$$

Here,

$$r_{lmn} = \left[(x_i - x_{jl})^2 + (y_i - y_{jm})^2 + (z_i - z_{jn})^2\right]^{1/2}, \quad (S6)$$

$$(x_{jl}, y_{jm}, z_{jn}) = [lL + (-1)^l x_j, mL + (-1)^m y_j, nL + (-1)^n z_j], \quad (S7)$$

and

$$q_{lmn,k} = \frac{1}{\varepsilon_k}\left[\frac{\varepsilon_k - \varepsilon_2}{\varepsilon_k + \varepsilon_2}\right]^{|l|+|m|+|n|}, \quad (S8)$$

with $\varepsilon_C = \varepsilon_s$ (the static dielectric constant of the perovskite material), $\varepsilon_Y = \varepsilon^*$ (ionic correction of the perovskite), and ε_2 the dielectric constant of the outer medium. The functions $f_{lmn,k}$ are given by:

$$f_{lmn,C}(\mathbf{r}_i,\mathbf{r}_j) = 1, \quad (S9)$$

$$f_{lmn,Y}(\mathbf{r}_i,\mathbf{r}_j) = \frac{1}{2}\left(\frac{\varepsilon_\infty}{\varepsilon_s}\right)^\gamma \left(e^{-\beta_i r_{lmn}} + e^{-\beta_j r_{lmn}}\right). \quad (S10)$$

The single-particle self-polarization potentials V_i^{self} are obtained from Eq. $S5$ by setting $\mathbf{r}_i = \mathbf{r}_j$, eliminating the $l = m = n = 0$ term (which represents the interaction of a charge carrier with itself), and dividing by 2 as corresponds to a self-energy.

B. Variational wave functions

We describe the exciton and biexciton ground states with variational functions built as the product of non-interacting functions (describing the confinement-defined limit) and appropriate correlation factors (describing the bulk limit). It is the interplay between confinement and carrier interactions that determines the optimal value of the (isotropic) variational parameters. For the exciton, the variational function reads:

$$\Psi_X = N_X \Phi_e(\mathbf{r}_e)\Phi_h(\mathbf{r}_h)\, e^{-Z_X r_{eh}}. \qquad (S11)$$

Here, N_X is the normalization factor, $\Phi_e(\mathbf{r}_e)$ and $\Phi_h(\mathbf{r}_h)$ are the non-interacting electron and hole (particle-in-box) states, which account for quantum confinement. The exponential term is a Slater correlation factor, which captures excitonic interactions. Z_X, the inverse of the exciton Bohr radius, is the parameter to optimize variationally.

For biexcitons, the variational function is[7-9]:

$$\Psi_{XX} = N_{XX}\ \Phi_e(\mathbf{r}_{e_1})\Phi_e(\mathbf{r}_{e_2})\Phi_h(\mathbf{r}_{h_a})\Phi_h(\mathbf{r}_{h_b})\ F_{XX}(r_{1a}, r_{1b}, r_{2a}, r_{2b}, r_{12}, r_{ab}). \qquad (S12)$$

Here, N_{XX} is the normalization factor, and F_{XX} is the correlation factor, described by:

$$F_{XX} = e^{-Z_{XX}\frac{(s_1+s_2)}{2}} \cosh\left(Z_{XX} Q \frac{t_1 - t_2}{2}\right) \times e^{Z_{XX}\frac{\beta r_{12}}{1+Z_{XX}\,\alpha r_{12}}} e^{Z_{XX}\frac{\beta r_{ab}}{1+Z_{XX}\alpha r_{ab}}}, \qquad (S13)$$

where $s_1 = r_{1a} + r_{1b}$, $s_2 = r_{2a} + r_{2b}$, $t_1 = r_{1a} - r_{1b}$ and $t_2 = r_{2a} - r_{2b}$, with r_{12}, r_{ab}, r_{1a}, r_{2a}, r_{2b} the interparticle distances. Z_{XX}, Q, β and α are the variational parameters to be optimized. The first term in Eq. $S13$ describes Slater correlation factors, analogous to that used for the exciton. Z can be seen as a scaling factor reflecting the strength of electron-hole attraction. Q allows for a non-symmetric interaction in the biexciton. In bulk, $Q = 0$, corresponds to a perfectly bound biexciton, with $r_{1a} = r_{1b} = r_{2a} = r_{2b}$, while $Q = 1$

corresponds to a pair of dissociated excitons. The last two exponentials in Eq. $S13$, involving β and α parameters, are Padé-Jastrow factors representing the carrier repulsion correlation.

C. Integration method

To integrate Hamiltonians $S1$ and $S2$ we rely on a variational Quantum Monte Carlo method[10,11]. The variational energy reads:

$$\langle E\rangle = \frac{\int d\mathbf{R}\ \Psi^*(\mathbf{R})H\Psi(\mathbf{R})}{\int d\mathbf{R}'\ \Psi^*(\mathbf{R}')\Psi(\mathbf{R}')} = \int d\mathbf{R}\ \ p(\mathbf{R})E_L(\mathbf{R}), \qquad (S14)$$

where $\Psi(R)$ is the trial function, with $\mathbf{R} = (\mathbf{r}_e, \mathbf{r}_h)$ for X and $\mathbf{R} = \left(\mathbf{r}_{e_1}, \mathbf{r}_{e_2}, \mathbf{r}_{h_a}, \mathbf{r}_{h_b}\right)$ for the biexciton, E_L the local energy:

$$E_L(\mathbf{R}) = \frac{\hat{H}\Psi(\mathbf{R})}{\Psi(\mathbf{R})}, \qquad (S15)$$

and $\mathrm{p}(R)$ the probability distribution:

$$p(\mathbf{R}) = |\Psi(\mathbf{R})|^2 / \int d\mathbf{R}'\, |\Psi(\mathbf{R}')|^2. \qquad (S16)$$

The Metropolis algorithm is used for important sampling[12]. Thus, particles are moved to different trial positions in a random walk. The new positions are accepted if the following condition is satisfied:

$$\frac{p(\mathbf{R}_{new})}{p(\mathbf{R}_{old})} > w, \qquad (S17)$$

where w is a random number homogeneously distributed between 0 and 1. For a sufficiently long calculation, this random walk allows to simplify Eq. $S14$ as:

$$\langle E\rangle \approx \frac{1}{N}\sum_{i=1}^{N} E_L(\mathbf{R}_i)\,, \qquad (S18)$$

where N is the number of (accepted) points R_i taken in the walk. An improved energy estimator has been proposed, which yields the same average but with smaller variance, by considering not only accepted steps, but also rejected ones[10]:

$$\langle E\rangle \approx \frac{1}{N}\sum_{i=1}^{N}\left[p(\mathbf{R}_{new})\,E_L(\mathbf{R}_{new}) + \left(1 - p(\mathbf{R}_{new})\right)E_L(\mathbf{R}_{old})\right], \qquad (S19)$$

with N here being the total number of attempted moves (whether accepted or not). We estimate the energy with Eq. $S19$ in our codes. For details (how the local energy is evaluated and the use of random walkers distributed among different CPU processes using OpenMP parallelization) see ref.[13].

D. Biexciton geometry

To determine the biexciton geometry, we calculate the most probable location of electrons and holes from the internal coordinates recorded during the Monte Carlo random walks, using Metropolis algorithm estimators[10,13]. Because of the permutation symmetry of $e_1 - e_2$ and $h_a - h_b$, the (averaged) interparticle distances obtained with this procedure satisfy $r_{1a} = r_{1b} = r_{2a} = r_{2b}$. However, the optimized wave function presents in most cases a nonzero value of the parameter Q. This is indicative that the biexciton is really formed by a pair of interacting excitons, with inter-exciton distances larger than intra-exciton ones. To draw forth this asymmetry, we calculate the interparticle distances avoiding permutation symmetry. For every step in the random walk, we choose a hole and estimate separately the distance with the closer electron and that with the more distant one. Electron–hole distances are then defined as:

$$r_{eh}^{min} = \frac{1}{2}\left[\min(r_{a1}, r_{a2}) + \min(r_{b1}, r_{b2})\right], \qquad (S20)$$

$$r_{eh}^{max} = \frac{1}{2}\left[\max(r_{a1}, r_{a2}) + \max(r_{b1}, r_{b2})\right]. \qquad (S21)$$

Even in the absence of excitonic correlations, this definition leads to $k = r_{eh}^{min}/r_{eh}^{max} < 1$, because there is always one particle farther than the other. To reduce this bias, we accept r_{eh}^{min} and r_{eh}^{max} in each step if:

$$\frac{r_{eh}^{min}}{r_{eh}^{max}} < w, \qquad (S22)$$

where w is a random number homogeneously distributed between 0 and 1. Otherwise, we state that particles are independent and then $r_{eh}^{min} = r_{eh}^{max} = (r_{a1} + r_{a2} + r_{b1} + r_{b1})/4$. To correct the residual bias, we renormalize using the electron−hole distances of uncorrelated biexciton. The latter is easily calculated setting $Z = 0$ in Ψ_{XX}. The coefficient:

$$k' = \frac{\left(r_{eh}^{max}/r_{eh}^{min}\right)}{\left(r_{eh}^{max}/r_{eh}^{min}\right)_{Z=0}} \qquad (S23)$$

is taken as the actual relation between r_{eh}^{min} and r_{eh}^{max}.

E. Radiative rates

Once the variational wavefunctions are optimized, the corresponding radiative recombination rates are calculated within the dipole approximation[14]. The exciton rate is given by[9]:

$$k_X^{rad} = \theta \left|\langle 0|\delta_{\mathbf{r}_e,\mathbf{r}_h}|\Psi_X\rangle\right|^2 = \theta\ N_X^2 \left(\frac{L}{2}\right)^6, \qquad (S24)$$

where θ is a constant of proportionality. In turn, the biexciton rate is given by:

$$k_{XX}^{rad} = \theta\, 3 \left|\left\langle \Psi_X \left|\delta_{\mathbf{r}_{e_2},\mathbf{r}_{h_b}}\right| \Psi_{XX}\right\rangle\right|^2 = \theta\, 3\, (N_X\, N_{XX}\, \mathcal{I})^2, \qquad (S25)$$

where the 3 factor arises because biexcitons triple the recombination channels allowed for excitons (see section II. G for details), considering the dark singlet state remains unoccupied, and:

$$\mathcal{I} = \int \Phi_e(\mathbf{r}_{e_1})^2\ \Phi_e(\mathbf{r}_{e_2})^2\ \Phi_h(\mathbf{r}_{h_a})^2\ e^{-\left(\frac{Z_{XX}}{2}+Z_x\right)r_{1a}} e^{-\frac{Z_{XX}}{2}r_{12}} e^{-\frac{Z_{XX}}{2}r_{2a}}$$

$$\times \cosh\left(\frac{Z_{XX}Q}{2}(r_{1a} - r_{12} - r_{2a})\right) e^{Z_{XX}\frac{\beta r_{12}}{1+Z_{XX}\alpha r_{12}}} e^{Z_{XX}\frac{\beta r_{2a}}{1+Z_{XX}\alpha r_{2a}}}\, d\mathbf{r}_{e_1}\, d\mathbf{r}_{e_2} d\mathbf{r}_{h_a}. \qquad (S26)$$

The integral $\mathcal{J}$ as well as the normalization factors N_X and N_{XX} are calculated numerically using Monte Carlo routines. The statistical error of each integral is minimized by averaging over up to 60 evaluations with different seeds.

F. Material Parameters for $CsPbBr_3$

We describe $CsPbBr_3$ QDs using the material parameters extracted in ref.[15]. Thus, bare electron and hole masses are $m_e^b = m_h^b = 0.234\, m_0$, with m_0 the free electron mass. The Fröhlich coupling constant is $\alpha = 2.1$, which gives polaronic masses $m_e = m_h = 0.316\, m_0$. The LO phonon energy is $E_{LO} = 18$ meV. From Eq. $S4$, the previous parameters yield polaron radii $l_e = l_h = 3$ nm (hereafter l_X). Relative static and dynamic dielectric constants inside the QD (ε_{QD}) are $\varepsilon_s = 16$ and $\varepsilon_\infty = 4.5$. The dielectric constant of the surrounding matrix (polystyrene) is set to $\varepsilon_{out} = 2.56$[16]. In order to retrieve the bulk X binding energy, $\Delta_X^{bulk} = 33$ meV[17,18], the Bajaj optimization parameter is set here to $\gamma = 3/13$. This value grants an optimal description of the polaronic exciton in bulk $CsPbBr_3$, and retrieves the emission resonances of excitons and trions in $CsPbBr_3$ QDs[8]. Using $\gamma = 3/5$, which is the (material-averaged) value proposed in ref.[5] for a number of different ionic crystals, underestimates the bulk exciton binding energy ($\Delta_X^{bulk}(\gamma = 3/5) = 19$ meV) and does not change the conclusions we derive here.

G. Ratio between exciton and biexciton recombination rates in the strong confinement limit

Eqs. $S11 - S12$ show the envelope function of exciton and biexciton, respectively. The complete wave function is the product of envelope and Bloch parts, $|X\rangle = \Psi_X |J_X\rangle$ and $|XX\rangle = \Psi_{XX} |J_{XX}\rangle$, with J_X and J_{XX} the total Bloch angular momenta. In the strong confinement limit, where correlations are weak ($Z_X \to 0, Z_{XX} \to 0$), the Bloch functions can be approximated as products of the constituting particles, $|J_X\rangle = |j_e\rangle |j_h\rangle$ and $|J_{XX}\rangle = |J_{2e}\rangle |J_{2h}\rangle$.

In $CsPbBr_3$, both conduction electrons and valence holes have envelope functions with a spin-like angular momentum j=1/2[19]. Consequently, for exciton, the Bloch angular momentum is $J_X = j_e \otimes j_h = 1 \oplus 0$. That is, the ground state described by $|X\rangle$ contains a fine structure with a triplet and a singlet. Electron-hole exchange interaction splits these sublevels energetically, with the singlet being usually under the triplet. Because a photon has angular

momentum 1, triplets are bright, while singlets are dark. If the lattice symmetry is not cubic, the bright triplet may experience additional splitting, but bright states remain bright, and the dark singlet remains dark[19]. In the biexciton case, however, there are two electrons and two holes. The two (indistinguishable) electrons give a total angular momentum $J_{2e} = j_{e1} \otimes j_{e2} = 1 \oplus [0]$, and the two (indistinguishable) holes give $J_{2h} = j_{h1} \otimes j_{h2} = 1 \oplus [0]$. Only the antisymmetric spin configuration is compatible with the (symmetric) double occupation of an orbital. Consequently, the biexciton ground state described by $|XX\rangle$ is formed through the combination of a singlet electron and a singlet hole, $J_{XX} = J_{2e} \otimes J_{2h} = [0]$. The different multiplicity of $|X\rangle$ (four-fold) and $|XX\rangle$ (one-fold) is responsible for different recombination rates in the strong confinement limit, as we show below.

Fig. S2 shows a sketch of the $|XX\rangle$ and $|X\rangle$ energy levels, relative to the closed shell configuration (all electrons in the valence band, labeled here as $|\emptyset\rangle$). Blue arrows represent optically allowed transitions. Note that, because both $|XX\rangle$ and $|\emptyset\rangle$ have J=0, transitions $|XX\rangle \rightarrow |X\rangle$ and $|X\rangle \rightarrow |\emptyset\rangle$ experience identical selection rules, with the J_X=0 state being forbidden.

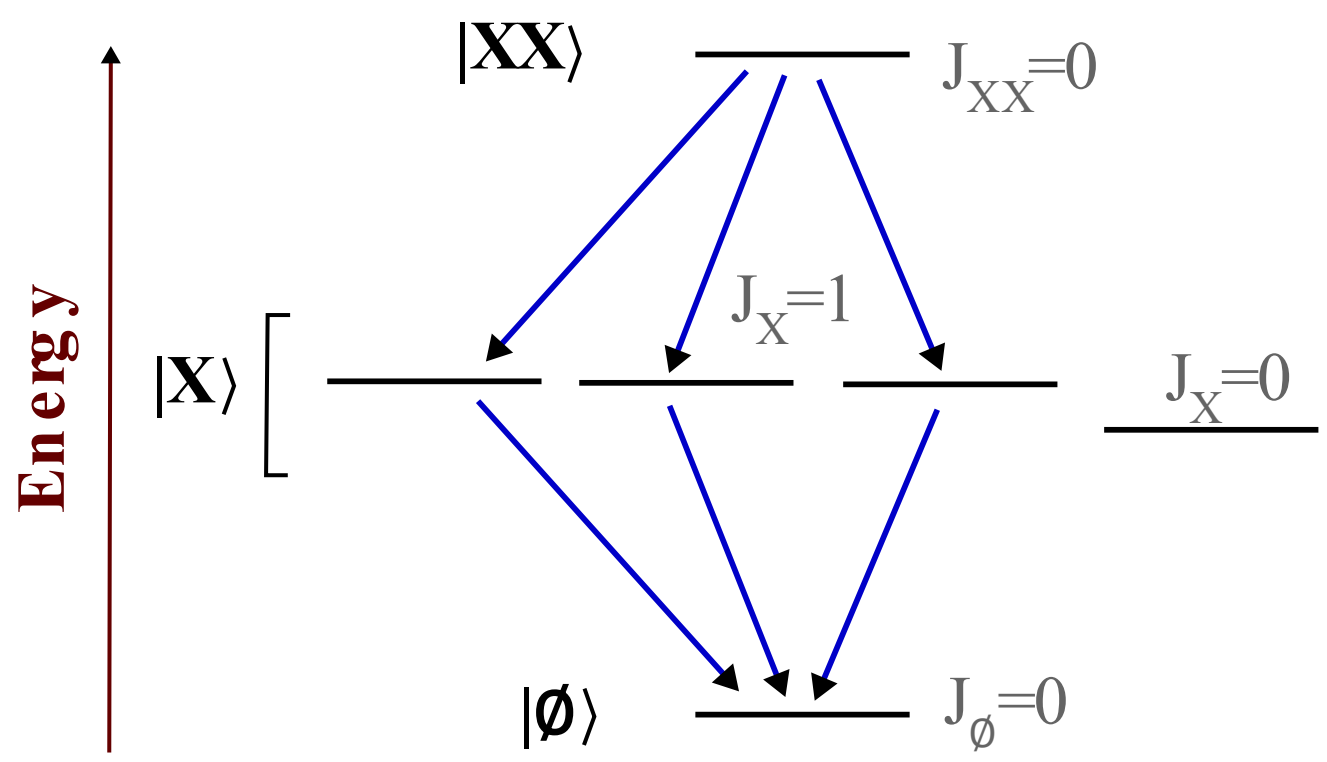

Fig. S2| One-photon optical transitions involving the biexciton and exciton ground state. Blue arrows show the optically allowed transitions. The singlet exciton state (J_x=0) is decoupled.

In the dipole approximation, optical transition rates between an initial state $|i\rangle$ and a final state $|f\rangle$, calculated through Fermi's golden rule are proportional to:

$$k_{i \rightarrow f} \propto \sum_{i,f} |\langle f|\hat{p}|i\rangle|^2 \, n_i \, (1 - n_f) \, \delta(h\nu - (E_f - E_i)) \qquad (S\,27)$$

where $\hat{p}$ is the momentum operator, n_i and n_f the occupation of initial and final states, and the delta function is the resonance condition. The momentum matrix element, upon factorization of envelope and Bloch parts, becomes $\langle f|\hat{p}|i\rangle = \langle \Psi_f|\Psi_i\rangle \langle j_f|\hat{p}|j_i\rangle$. Eq. S27 can then be rewritten under resonance condition as:

$$k_{i\to f} \propto \sum_{i,f} \left|\langle \Psi_f|\Psi_i\rangle \langle j_f|\hat{p}|j_i\rangle\right|^2 n_i \; (1-n_f) \qquad (S28)$$

The Bloch part imposes the selection rules discussed above: only initial and final states differing by $\Delta J = \pm 1$ are allowed, (that is, transitions involving the triplet exciton states). The envelope part is what we have computed in Eqs. (S24) and (S25). Assuming that the final states are empty (n_f =0), and the initial state is occupied (n_i =1), the $|XX\rangle \to |X\rangle$ transition becomes:

$$k_{XX\to X} \propto 3|\langle \Psi_X|\Psi_{XX}\rangle|^2 \qquad (S29)$$

which is the expression we gave in Eq.(S25). For the $|X\rangle \to |\emptyset\rangle$ transition, there is experimental evidence that only the three triplet (bright) states are occupied prior to radiative recombination[20,21]. Then, there are three transitions from states with initial occupation ni =1/3 each:

$$k_{X\to\emptyset} \propto |\langle \emptyset|\Psi_X\rangle|^2 \qquad (S30)$$

which is the expression we gave in Eq. S24. In the strong confinement regime, $\langle \Psi_X|\Psi_{XX}\rangle = \langle \emptyset|\Psi_X\rangle$. Then, $k_{XX\to X}/k_{X\to\emptyset} = 3$. In small $CsPbBr_3$ nanocrystals, the dark exciton is weakly coupled to the bright one[21]. If this is the case, $k_{XX\to X}/k_{X\to\emptyset}$ may increase up to 4. As shown in **Fig. S3**, this change would require larger QD sizes to reach $k_{XX\to X}/k_{X\to\emptyset} = 1$, but the qualitative behavior is the same.

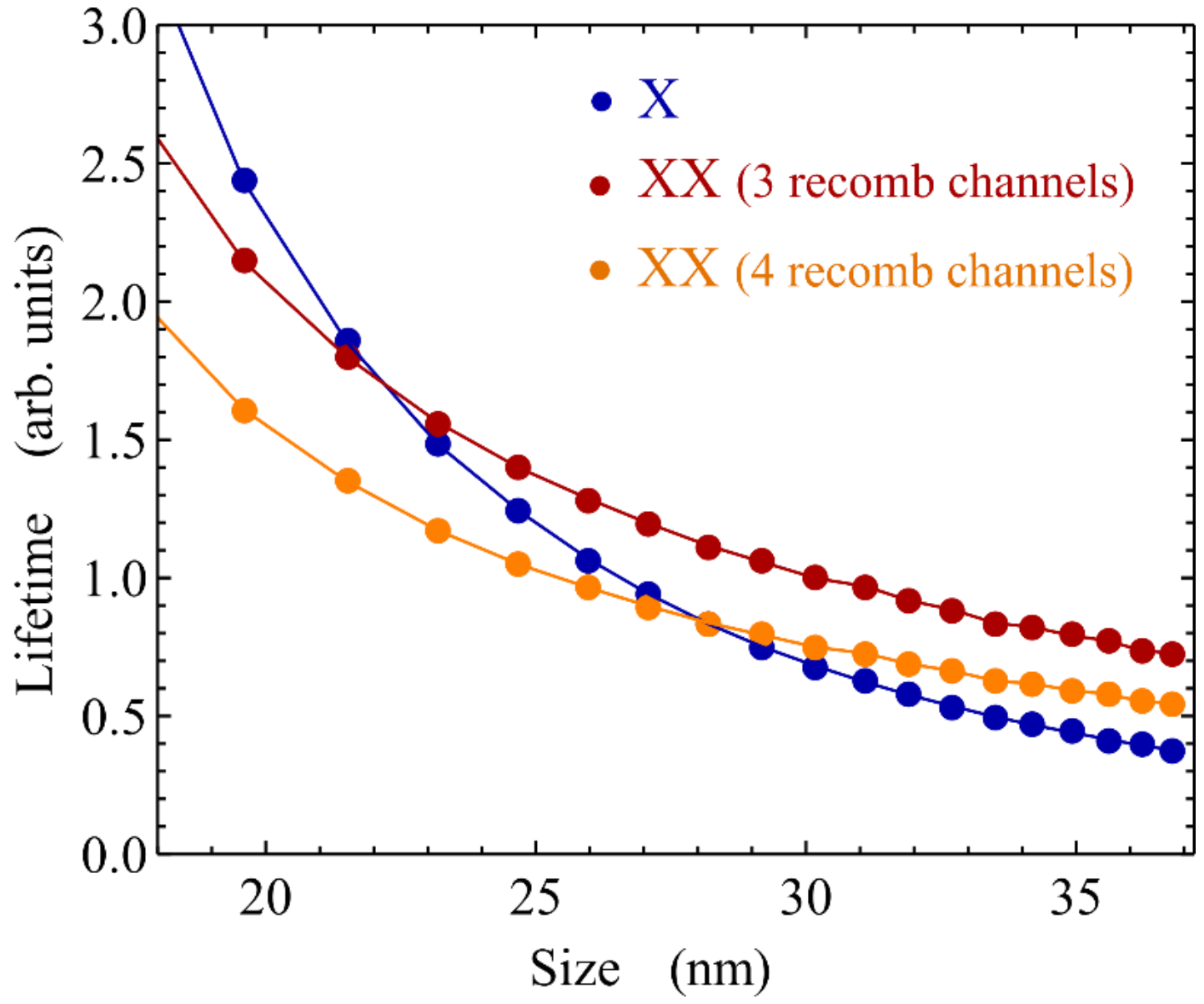

Fig. S3| Relative radiative lifetimes of exciton and biexciton, assuming the biexciton has 3 (as in the main text) or 4 more recombination channels than the exciton.

III. Further experimental details

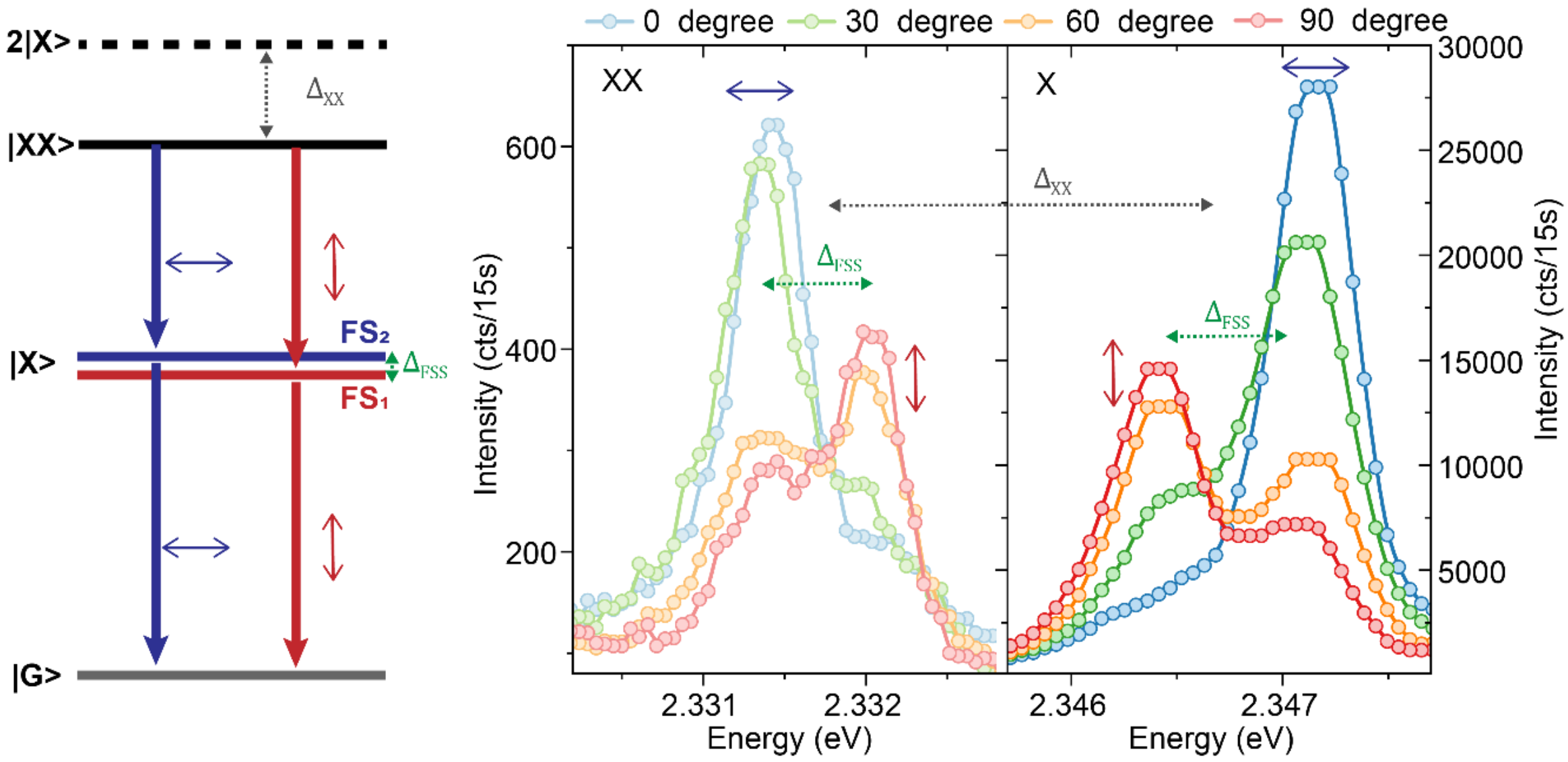

Fig. S4| Polarization-resolved fine-structures (doublet) of exciton and biexciton in a 25.9 nm $CsPbBr_3$ QD at 4 K.

To fully resolve the fine-structures (FS) of the exciton, phonon replicas and biexciton, the PL spectrum is fitted with multi-Lorentzian functions as follows:

$$I_{tot}(E) = I_X(E) + \sum_{j=1,2,3} I_{OP_j,X}(E) + I_{XX}(E) + \sum_{j=1,2} I_{OP_j,XX}(E) + c \qquad (S31)$$

where $I_X(E)$ and $I_{XX}(E)$ are the exciton and biexciton emission, respectively, $I_{OP_j,X}(E)$ and $I_{OP_j,XX}(E)$ represent different phonon replicas of exciton and biexciton, respectively, and c is the background. For the exciton term, we fit as follows:

$$I_X(E) = \sum_{i=1,2,3} \frac{2 \times I_{FS_i}}{\pi} \frac{w_{FS_1,X}}{4 \times (E - E_{exc} - \Delta_i)^2 + w_{FS_1,X}^2} \qquad (S32)$$

where $\Delta_i = E_i - E_1$ is the splitting energy between FS. For the phonon replicas of the exciton, we fit as follows:

$$I_{OP_j,X}(E) = \sum_{i=1,2,3} \frac{2 \times S_{j,X} \times I_{FS_i}}{\pi} \frac{w_{FS_i,OP_j,X}}{4 \times (E - E_{exc} - E_{OP_j} - \Delta_i)^2 + w_{FS_1,OP_j,X}^2} \qquad (S33)$$

where the $S_{j,X}$ is the coupling strength for the phonon mode OP_j. For the biexciton, we fit as follows:

$$I_{XX}(E) = \sum_{i=1,2,3} \frac{2\times R_{XX}\times I_{FS_i}}{\pi} \frac{w_{FS_i,XX}}{4\times(E-E_{exc}-\Delta_{XX}+\Delta_i)^2 + w^2_{FS_i,XX}} \quad (S34)$$

where R_{XX} represents the intensity ratio of biexciton to exciton for the fine-structure state decaying through the same intermediate state. For the phonon replicas of the biexciton, we fit as follows:

$$I_{OP_j,XX}(E) = \sum_{i=1,2} \frac{2\times R_{XX}\times S_{j,XX}\times I_{FS_i}}{\pi} \frac{w_{FS_i,OP_j,XX}}{4\times(E-E_{exc}-\Delta_{XX}-E_{OP_j}+\Delta_i)^2 + w^2_{FS_i,OP_j,XX}} \quad (S35)$$

Throughout our spectral fits to the different emitting species, the relative intensity distribution and splitting energies among the fine structures are fixed. The obtained spectral fits are plotted in **Fig. 2d** and **Fig. S5a-b**.

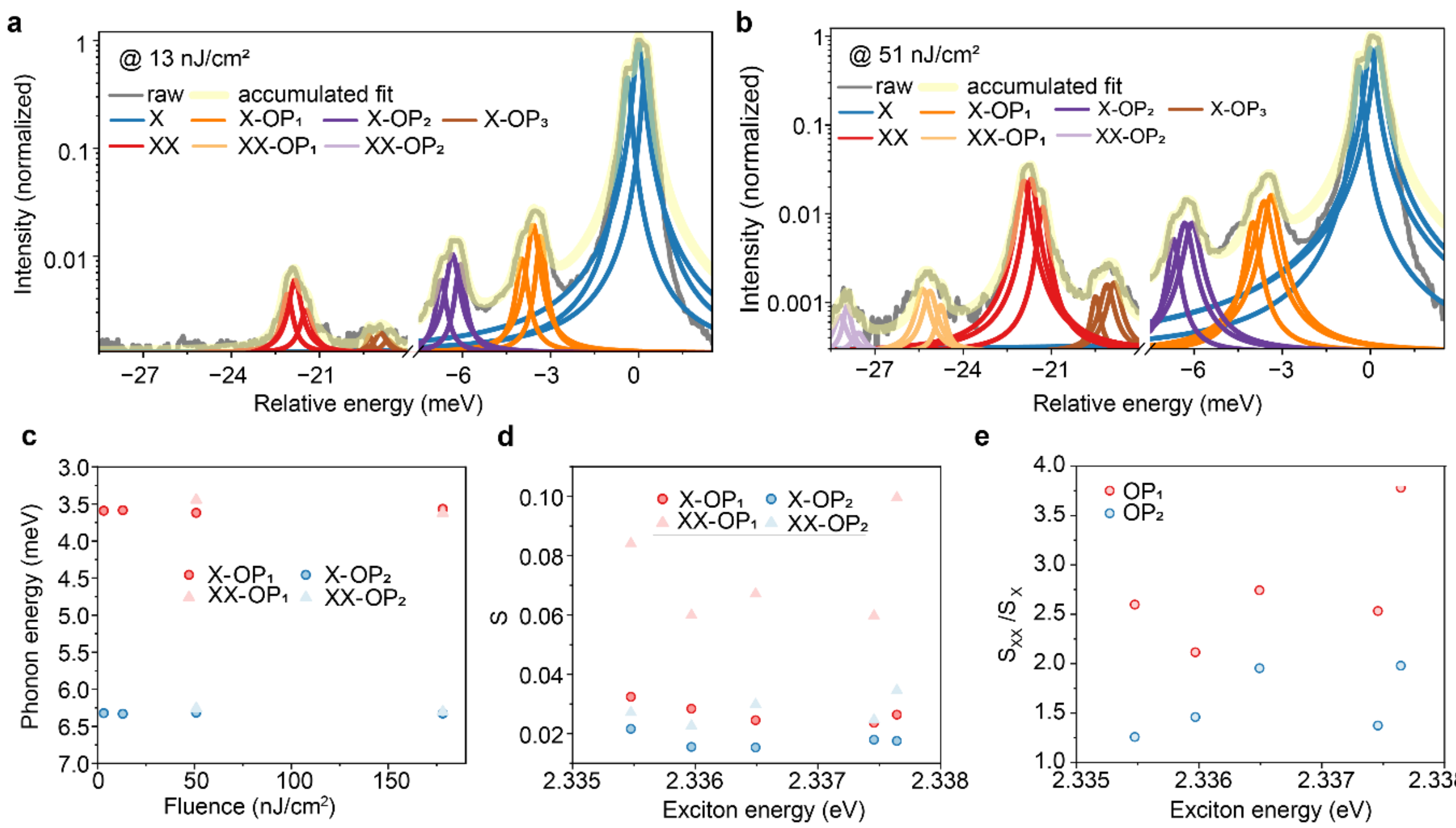

Fig. S5| X-phonon and XX-phonon coupling in individual CsPbBr$_3$ QDs at 4 K. a,**b**, PL spectrum of the QD plotted in **Fig. 2** at a laser fluence of 13 nJ/cm^2 and 51 nJ/cm^2, respectively. **C**, Phonon energies of the OP$_1$ and OP$_2$ mode for exciton and biexciton. **D**, Coupling strength of the exciton and biexciton to the OP$_1$ and OP$_2$ phonon mode at different

exciton energies. **E**, Coupling strength ratio S_{XX}/S_X for the OP_1 and OP_2 mode for several individual QDs; the coupling strength ratio is especially high for the OP_1 mode.

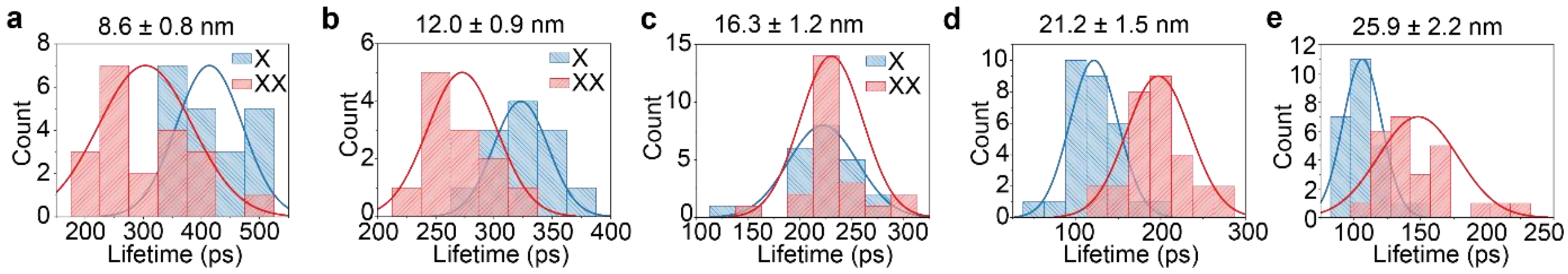

Fig. S6| Distribution of radiative lifetimes of exciton and biexciton at 4 K in individual QDs obtained from five QD batches with increasing mean QD size. The unusual regime of a biexciton decaying slower than a exciton is reached for QDs larger than about 17 nm.

We further performed time-resolved PL measurements, at 4 K, on an ensemble film of the 25.9 nm quantum-dot batch using a streak camera with a temporal resolution of approximately 1 ps. The results are presented in **Fig. S7** and provide independent support for our interpretation. As the excitation power increases, a second emission peak gradually appears on the low-energy side of the exciton emission (**Fig. S7a-d**). The energy separation between the two peaks is approximately 15 meV (**Fig. S7d**), which agrees well with previously reported biexciton binding energies determined from single-QD measurements[16,22,23]. This agreement allows us to confidently assign the lower-energy peak to biexciton emission. We then extracted the time-resolved PL decay curves of both the exciton and biexciton peaks (**Fig. S7f**). Mono-exponential fitting reveals that the biexciton exhibits a longer lifetime than the exciton. The extracted lifetime ratio, shown in the inset of **Fig. S7f**, is averagely around 1.55, in excellent agreement with the values obtained from our single-quantum-dot measurements (**Fig. 3c**). These independent ensemble measurements therefore provide additional experimental evidence that fully supports our central conclusion that the biexciton lifetime exceeds the exciton lifetime in these large $CsPbBr_3$ QDs.

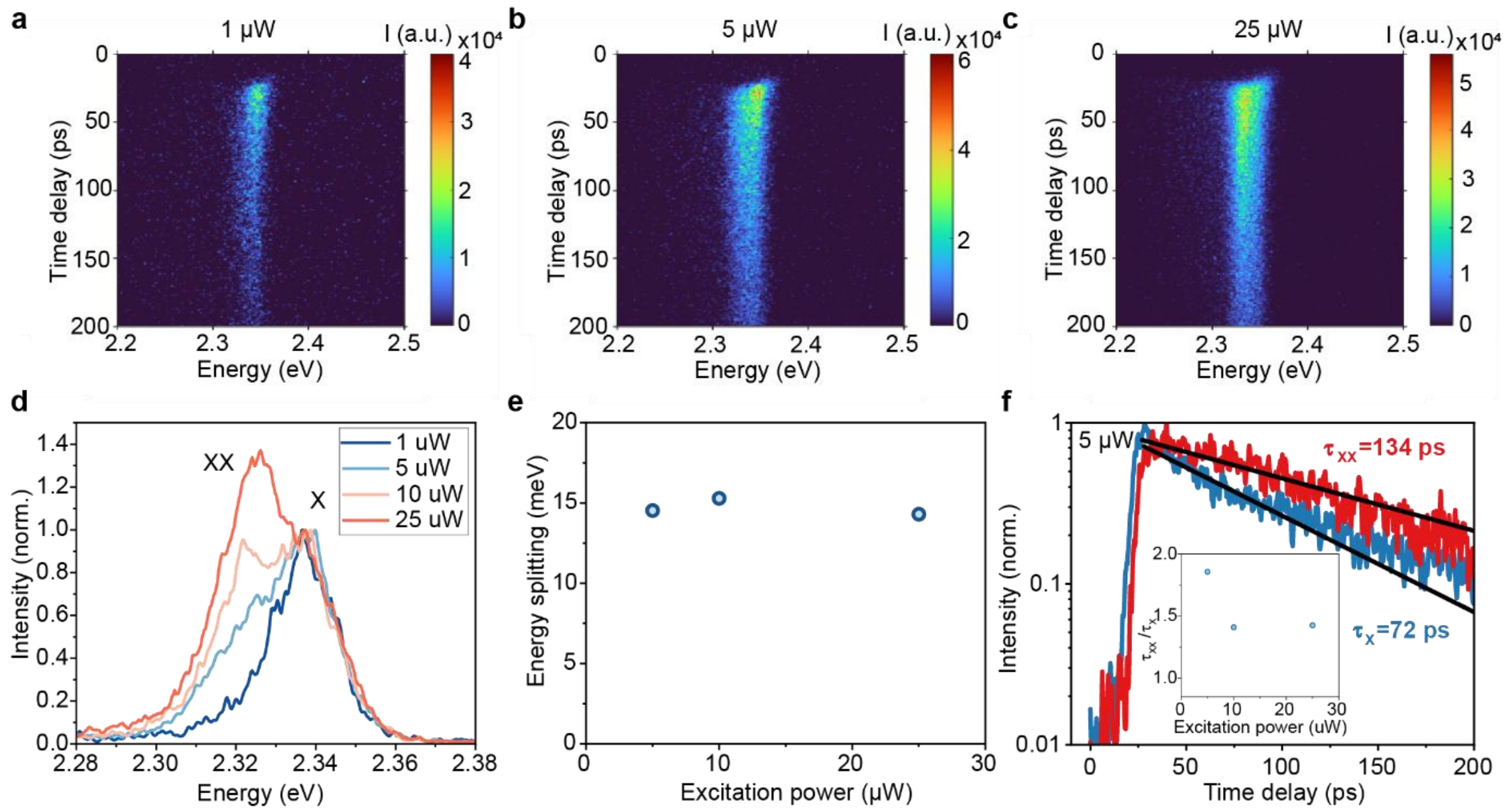

Fig. S7 | Time-resolved PL study of an ensemble film of the 25.9 nm quantum-dot batch at 4 K. a-c, The time-resolved spectra of one ensemble film of the 25.9 nm quantum-dot batch under different excitation powers. **d,** PL spectra of the ensemble film measured under different excitation powers. **e,** Energy separation between the exciton and biexciton emission peaks as a function of excitation power. **f,** Time-resolved photoluminescence (TRPL) decay curves recorded using a streak camera with a temporal resolution of approximately 1 ps. Inset: Ratio of the biexciton to exciton radiative lifetimes extracted at different excitation powers.

Details of the data analysis of the TRPL curves.

Both exciton and biexciton exhibit near-unity PLQY, as discussed in the main text. Therefore, their recombination dynamics are expected to be predominantly radiative, and we used a mono-exponential model to describe the corresponding decay curve. The extracted lifetime thus represents the characteristic decay duration required for the PL intensity to decrease to 1/e of its initial value following photoexcitation, as stated in the manuscript. Importantly, the lifetime extraction was performed using a mono-exponential fit over the early-to-intermediate time window where the signal-to-noise ratio is highest and where the decay exhibits a clean single-exponential behavior (see **Fig. 3**). For small QDs with relatively long decay traces, the fitting range typically spans ~90–95% of the total intensity decay. In this range, the agreement between the fit and the experimental data is excellent. The long-time tail, where constant background noise becomes comparable to the PL signal, is intentionally excluded to avoid systematic overestimation of the lifetime. For large QDs, a long decay component observed in the decay curves is primarily caused by the instrument response function (IRF) and not associated with intrinsic recombination dynamics of excitons or biexcitons. It is therefore

excluded from our lifetime analysis. To maintain a consistent fitting model across QDs of different sizes, we fitted the decay curves of large QDs with a mono-exponential model as well, restricting the fitting window prior to the IRF-limited region. Within this window, the decay behavior is well described by a single exponential.

IV. Further theoretical results

A. Robustness against polaronic model and parameters

The unconventional biexciton dynamics reported in this study is not directly related to polaronic effects. In fact, theoretical predictions of a X-XX lifetime reversal had been put forward by some of us in CdSe nanoplatelets[9], whose lattice is stiffer than that of metal halide perovskites. Yet, in this section we explore the effect of the specific polaronic description used in the modeling. The biexciton binding energies in **Fig.2b** of the main text were calculated using a Bajaj potential for the dielectric screening (Eq. S3). It was concluded that $l_{XX} > l_X$ is needed to reproduce the experimental measurements. Here, we use the original Haken potential instead[6]:

$$V_c^{bulk,Haken}(\mathbf{r}_i, \mathbf{r}_j) = \frac{q_i q_j}{\varepsilon_s\, r} + \frac{q_i q_j}{\varepsilon^*\, r}\, \frac{e^{-\beta_i r} + e^{-\beta_j r}}{2}, \qquad (S36)$$

which has the advantage of retrieving the due dielectric constant in the limits of $l_X=0$ (ε_s) and $l_X=\infty$ (ε_∞). In this case, in order to retrieve the bulk exciton binding energy of $CsPbBr_3$, Δ_X^{bulk}, we set $\varepsilon_\infty = 5.2$ and $E_{LO} = 19.2$ meV. The rest of parameters remain the same. **Fig.S7** shows that the binding energies of confined biexciton are almost the same for Bajaj and Haken potentials. In both cases, $l_{XX} > l_X$ is needed to match the large binding energies of experiments.

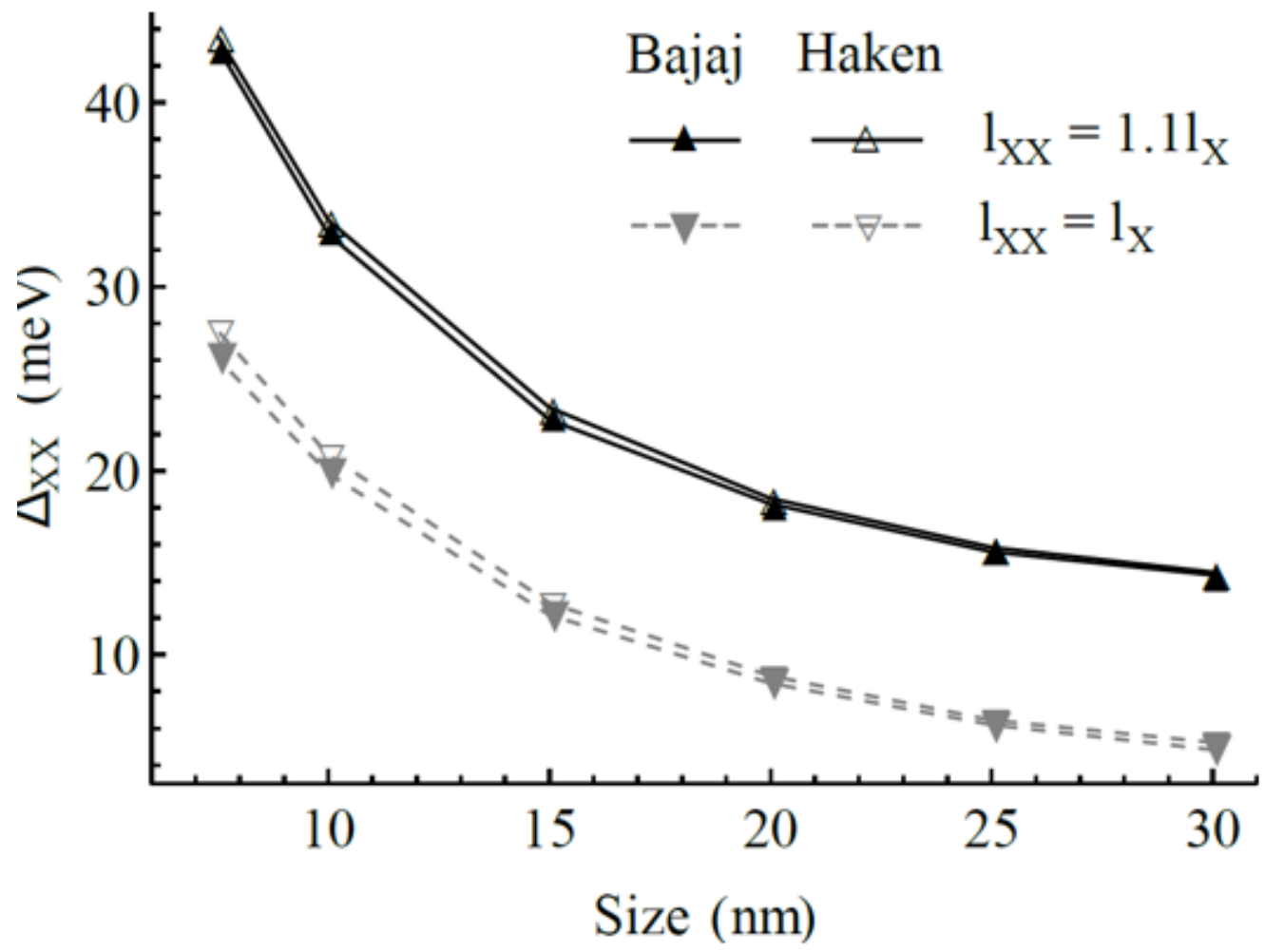

Fig. S8| Comparison of the biexciton binding calculated using Bajaj and Haken potentials of polaronic interaction, as a function of the QD size.

Fig. S9a shows that the radiative decay of exciton and biexciton calculated with a Haken potential is very similar to that obtained with Bajaj's potential (**Fig. S9b** or **Fig. 3b** in the main text). **Fig. S9b** further shows that the slowdown of the biexciton rate in large QDs remains if we keep $l_{XX} = l_X$, rather than setting $l_{XX} = 1.1 l_X$ to fit Δ_{XX}.

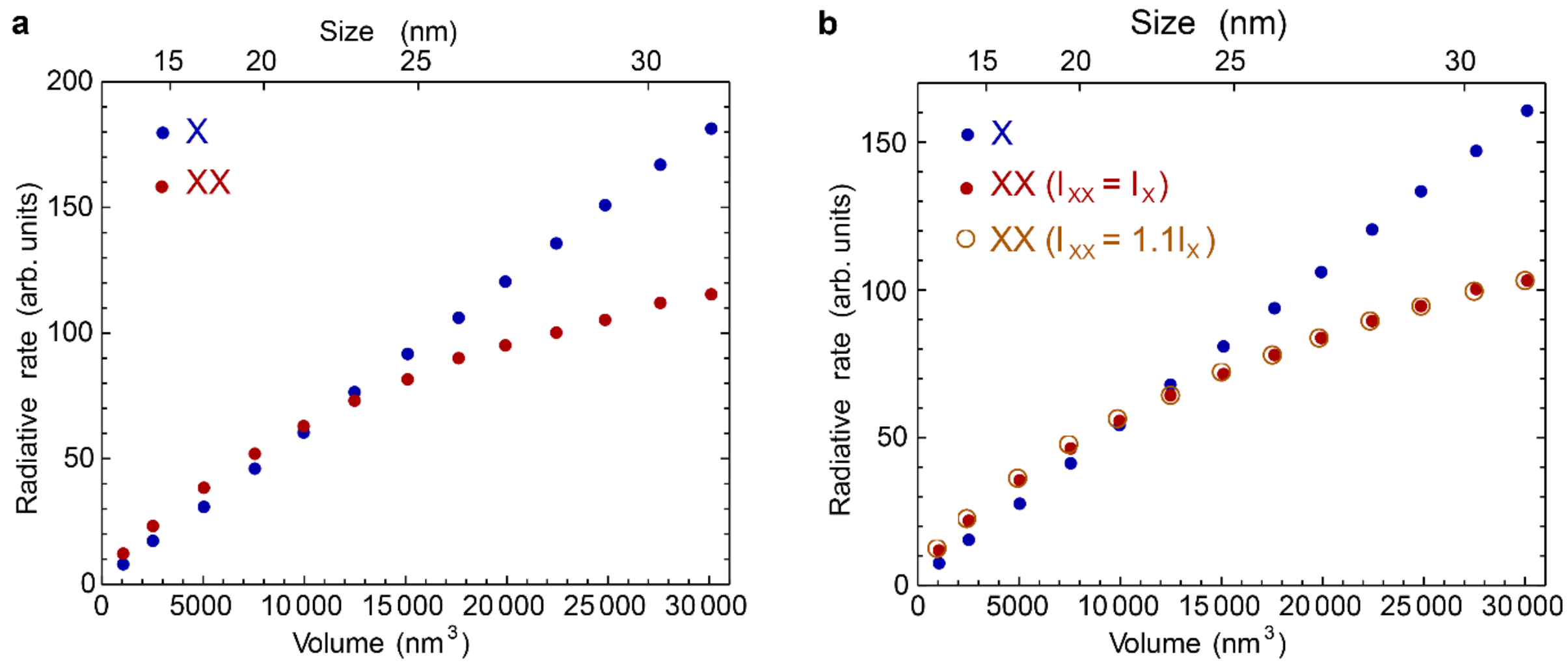

Fig. S9| Exciton and biexciton radiative rates calculated for different QD sizes. **a,** results with a Haken potential. **b,** results with a Bajaj potential and different values of the XX polaronic radius.

B. Influence of different types of correlation

The variational parameters in F_{XX} -Eq. S13- account for different many-body effects in the biexciton. Z_{XX} describes the electron-hole correlation, α and β the electron-electron and hole-hole correlation, and Q the degree of dissociation of biexciton.

Fig.S10 shows that α and β have minor influence on the X-XX radiative rate reversal. When Q is fixed to 1 (dissociated biexciton, grey symbols), the biexciton rate is systematically greater than that of exciton, no matter if we switch off ($\alpha = \beta = 0$) or on ($\alpha = \beta = 1$) repulsive correlations. The same conclusion holds for different values of Q. Thus, when we optimize Q for each QD size (red symbols), the biexciton rate bends down under the exciton rate, with moderate influence of α and β.

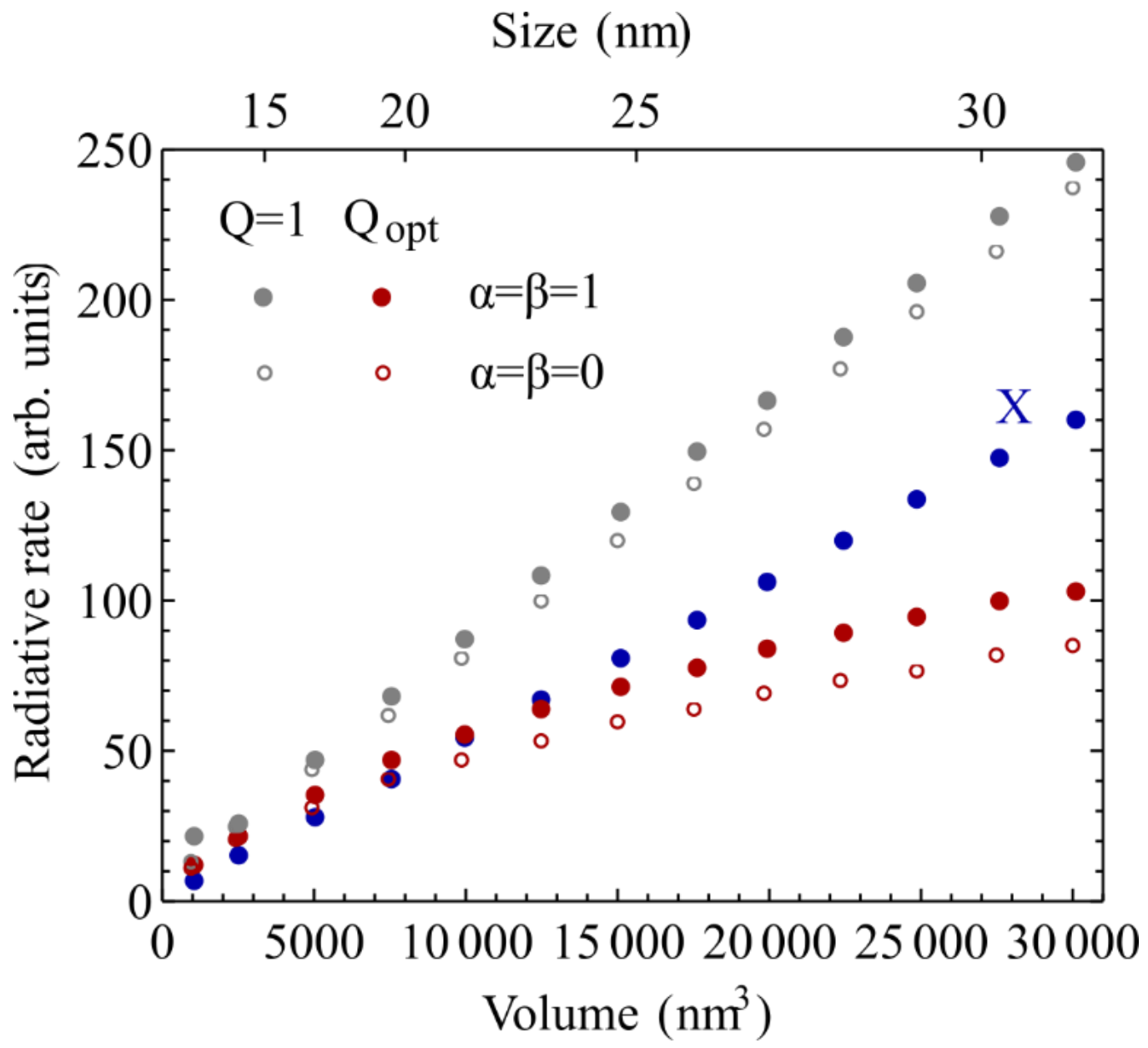

Fig. S10| Exciton and biexciton radiative rates calculated for different QD sizes, in the absence and presence of repulsive correlations. $Q = 1$ series correspond to dissociated biexciton. Q_{opt} series correspond to the lowest-energy (optimized) biexciton.

Fig.3d in the main text and **Fig.S10** suggest Q is the key parameter reducing the biexciton decay rate as compared to exciton. A direct connection between Q and electron-hole correlations can be established if we expand the hyperbolic cosine in F_{XX}:

$$e^{-Z_{XX}\frac{(s_1+s_2)}{2}} \cosh\left(Z_{XX} Q \frac{t_1 - t_2}{2}\right) =$$

$$= e^{-\left(Z^{intra}(r_{1a}+r_{2b})+Z^{inter}(r_{1b}+r_{2a})\right)} + e^{-\left(Z^{inter}(r_{1a}+r_{2b})+Z^{intra}(r_{1b}+r_{2a})\right)} \quad (S37)$$

Here, we have defined $Z^{intra} = Z_{XX}(1+Q)/2$ and $Z^{inter} = Z_{XX}(1-Q)/2$. One can identify the resulting exponentials of Eq. (S37) as X-like Slater correlation factors. The first summand, $e^{-Z^{intra}(r_{1a}+r_{2b})}$, describes a biexciton formed by two independent excitons: one where electron *1* pairs up with hole *a*, and another where electron *2* pairs up with hole *b*. Each of them is bound by Z^{intra}. At the same time, $e^{-Z^{inter}(r_{1b}+r_{2a})}$ describes cross-correlations between the two excitons: electron *1* with hole *b* and electron *2* with hole *a*. This is the inter-X correlation. Because $Q \geq 0$, intra-X correlations prevail over inter-X ones, $Z^{intra} \geq Z^{inter}$. The second summand in (S33) is the consequence of particle indistinguishability between the two electrons and the two holes.

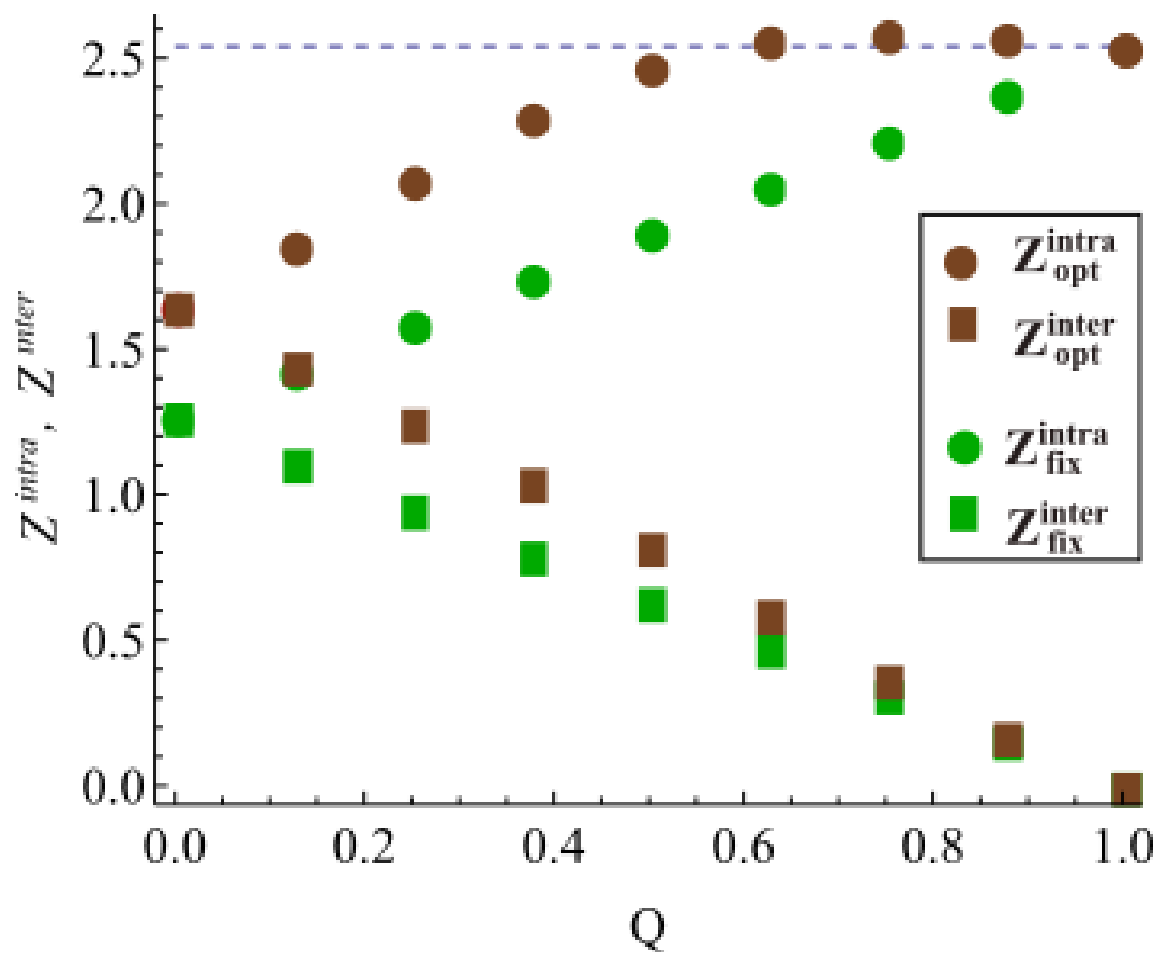

Fig. S11| Intra-X and Inter-X correlation factors as a function of Q in a superradiant QD. Brown symbols: Z is optimized for every Q. Green symbols: Z is fixed to the exciton value (dashed line). The QD has 36.8 nm lateral size. In the figure, $\alpha = \beta = 1$. The dashed line gives Z_X, for comparison.

Fig. S11 studies the dependence of $Z^{intra}$and Z^{inter} on Q. This is done for two series, a biexciton whose Z is artificially fixed to $Z_{XX} = Z_X$ (green symbols), and another where it is optimized variationally for every Q (brown symbols). In both cases, when Q increases (biexciton dissociates), intra-X correlations become stronger, at the expense of weakening inter-X ones. An important observation is that, when Z_{XX} is variationally optimized (brown symbols), Z^{intra} saturates to a maximum value of $Z^{intra} = Z_X$ (see asymptotic convergence to Z_X for $Q \geq 0.6$, represented in the figure by the horizontal, dashed line). This result indicates that the intra-X correlation of biexcitons never exceeds that of individual excitons. Yet, the biexciton radiative rate continues to increase beyond this point (see **Fig. 3d** for $Q \geq 0.6$). The conclusion is that the biexciton radiative rate is not set by Z^{intra}. Rather, it is set by Z^{inter}. The latter shows a monotonic behavior with Q, in line with the decay rate of **Fig. 3d**. The larger Q, the weaker the inter-X correlation, and the faster the biexciton recombination.

REFERENCES

1 Akkerman, Q. A. *et al.* Controlling the nucleation and growth kinetics of lead halide perovskite quantum dots. *Science* **377**, 1406–1412 (2022).

2 Morad, V. *et al.* Designer phospholipid capping ligands for soft metal halide nanocrystals. *Nature* **626**, 542–548 (2024).

3 Takagahara, T. Effects of dielectric confinement and electron-hole exchange interaction on excitonic states in semiconductor quantum dots. *Phys. Rev. B* **47**, 4569 (1993).

4 Movilla, J. L., Planelles, J. & Climente, J. I. Excitons in metal halide perovskite nanoplatelets: an effective mass description of polaronic, dielectric and quantum confinement effects. *Nanoscale Adv.* **5**, 6093–6101 (2023).

5 Bajaj, K. Effect of electron-phonon interaction on the binding energy of a wannier exciton in a polarizable medium. *Solid State Commun.* **15**, 1221–1224 (1974).

6 Baranowski, M. & Plochocka, P. Excitons in metal-halide perovskites. *Adv. Energy Mater.* **10**, 1903659 (2020).

7 Climente, J. I., Movilla, J. L. & Planelles, J. Electronic structure of biexcitons in metal halide perovskite nanoplatelets. *J. Phys. Chem. Lett.* **15**, 7379–7386 (2024).

8 Movilla, J. L., Planelles, J. & Climente, J. I. Binding energy of polaronic trions and biexcitons in $CsPbBr_3$ nanocrystals. *Phys. Rev. B* **111**, 155304 (2025).

9 Macias-Pinilla, D. F., Planelles, J. & Climente, J. I. Biexcitons in CdSe nanoplatelets: geometry, binding energy and radiative rate. *Nanoscale* **14**, 8493–8500 (2022).

10 Ceperley, D., Chester, G. V. & Kalos, M. H. Monte Carlo simulation of a many-fermion study. *Phys. Rev. B* **16**, 3081 (1977).

11 Needs, R. J., Towler, M. D., Drummond, N. D. & Ríos, P. L. Continuum variational and diffusion quantum Monte Carlo calculations. *J. Phys. Condens. Matter.* **22**, 023201 (2009).

12 Koonin, S. E. *Computational physics: Fortran version*. (CRC Press, 2018).

13 Planelles, J. & Climente, J. I. A simple variational quantum Monte Carlo-effective mass approach for excitons and trions in quantum dots. *Comput. Phys. Commun.* **261**, 107782 (2021).

14 Jacak, L., Hawrylak, P. & Wojs, A. *Quantum dots*. (Springer Science & Business Media, 2013).

15 Baranowski, M. *et al.* Polaronic mass enhancement and polaronic excitons in metal halide perovskites. *ACS Energy Lett.* **9**, 2696–2702 (2024).

16 Zhu, C. *et al.* Many-body correlations and exciton complexes in $CsPbBr_3$ quantum dots. *Adv. Mater.* **35**, 2208354 (2022).

17 Zhu, C. *et al.* Single-Photon Superradiance in Individual Caesium Lead Halide Quantum Dots. *Nature* **626**, 535–541 (2024).

18 Yang, Z. *et al.* Impact of the halide cage on the electronic properties of fully inorganic cesium lead halide perovskites. *ACS Energy Lett.* **2**, 1621–1627 (2017).

19 Sercel, P. C. *et al.* Exciton fine structure in perovskite nanocrystals. *Nano letters* **19**, 4068–4077 (2019).

20 Tamarat, P. *et al.* The dark exciton ground state promotes photon-pair emission in individual perovskite nanocrystals. *Nature communications* **11**, 6001 (2020).

21 Rossi, D. *et al.* Size-dependent dark exciton properties in cesium lead halide perovskite quantum dots. *The Journal of Chemical Physics* **153** (2020).

22 Tamarat, P. *et al.* Universal scaling laws for charge-carrier interactions with quantum confinement in lead-halide perovskites. *Nature Communications* **14**, 229 (2023).

23 Cho, K. *et al.* Size dependence of trion and biexciton binding energies in lead halide perovskite nanocrystals. *ACS nano* **18**, 5723–5729 (2024).
